# Yeasts from Chinese strong flavour *Daqu* samples: isolation and evaluation of their potential for fortified *Daqu* production

**DOI:** 10.1186/s13568-021-01337-y

**Published:** 2021-12-24

**Authors:** Shunchang Pu, Yu Zhang, Ning Lu, Cuie Shi, Shoubao Yan

**Affiliations:** 1 Department of Biology and Food Engineering, Bozhou University, Bozhou, 236800 Anhui People’s Republic of China; 2 School of Life Science, Huainan Normal University, Huainan, 232001 Anhui People’s Republic of China

**Keywords:** Yeast, Fortified *Daqu*, Volatile flavor compounds, Fermentation

## Abstract

In total, 16 yeast were isolated from Chinese strong flavour *Daqu* samples and underwent RAPD analysis and identification. Totally, 11 different species were identified among these isolates including *Saccharomyces cerevisiae, Hanseniaspora vineae*, *Pichia kluyveri*, *Trichosporon asahii*, *Wickerhamomyces anomalus*, *Kluyveromyces lactis*, *Yarrowia lipolytica*, *Wickerhamomyces mori*, *Galactomyces geotrichum*, *Dabaryomyces hansenii*, and *Saccharomyces kudriavzevii*. To understand the impact of these yeast strains on the quality and flavour of *Daqu*, we then assessed volatile compounds associated with *Daqu* samples fermented with corresponding strains. These analyses revealed strain YE006 exhibited the most robust ability to produce ethanol via fermentation but yielded relatively low quantities of volatile compounds, whereas strain YE010 exhibited relatively poor fermentation efficiency but produced the greatest quantity of volatile compounds. These two yeast strains were then utilized in a mixed culture to produce fortified *Daqu*, with the optimal inoculum size being assessed experimentally. These analyses revealed that maximal fermentation, saccharifying, liquefying, and esterifying power as well as high levels of volatile compounds were achieved when using a 2% inoculum composed of YE006/YE010 at a 1:2 (v/v) ratio. When the liquor prepared using this optimized fortified *Daqu* was compared to unfortified control *Daqu,* the former was found to exhibit significantly higher levels of flavour compounds and better sensory scores. Overall, our findings may provide a reliable approach to ensuring *Daqu* quality and improving the consistency and flavour of Chinese strong-flavour liquor through bioaugmentation.

**Supplementary Information:**

The online version contains supplementary material available at 10.1186/s13568-021-01337-y.

## Introduction

Chinese strong-flavour liquor is a traditional distilled alcoholic beverage that plays a central role in many aspects of Chinese culture. In addition to being widely consumed in China, this liquor is increasingly popular in East Asia owing to its unique flavour and brewing approach. Chinese strong-flavour liquor is produced via the spontaneous solid-state fermentation of a mixture of sorghum, wheat, and/or rice through a process dependent upon *Daqu* manufacture and grain fermentation (Yan et al. 2015). *Daqu* is a saccharifying and fermenting agent utilized in the brewing of Chinese strong-flavour liquor, lending the beverage its characteristic flavour properties (Zheng et al. 2011). *Daqu* is a source of key crude enzymes, microbes, and aroma precursors that ultimately govern the composition of the final liquor product (Yan et al. 2019). Given its central role in the manufacture of Chinese strong-flavour liquor, Chinese brewers use summarize its importance with the expression “better *Daqu*, better liquor” (Ming et al. 2015).

Chinese strong-flavour *Daqu* is traditionally composed of wheat, barley, and/or peas in a three-stage preparation process (Zheng et al. 2011). First, materials are ground, mixed, and shaped, after which solid-state fermentation at a controlled temperature occurs, followed by drying and ripening. In most cases, manufacturers do not selectively introduce any specific microorganisms to alter the *Daqu* fermentation process. However, as traditional *Daqu* production involves the utilization of non-autoclaved raw materials in an open environment, the microbial communities that facilitate the eventual solid-state fermentation process are sensitive to changes in moisture, temperature, pH, and acidity such that this process cannot be reliably controlled. This has the potential to yield *Daqu of* inconsistent quality, resulting in higher production costs and reduced fermentation power that can compromise the liquor production process.

Yeast species are the primary microorganisms responsible for the fermentation activity of *Daqu,* contributing to the generation of ethanol as well as the production of a range of volatile flavour compounds including esters, ketones, alkenes, and phenols. The high temperatures often used in the production of *Daqu* (50–55 ℃), however, can inhibit the fermentative activity and growth of these yeasts, explaining the relatively weak fermentation activity of Chinese strong-flavour *Daqu.* Modern research has led food producers to increasingly utilize select functional microorganisms to accelerate and guide fermentation processes to ensure a more consistent and higher quality final product. For example, Lactobacillus, Pediococcus, Leuconostoc, and Staphylococcus starters have been used to support sausage and vegetable fermentation (Li et al. 2017), while *Bacillus subtilis*, *B. licheniformis*, and *B. amyloliquefaciens* have been isolated and leveraged to reliably ferment traditional Chinese sesame-flavoured liquor (Li et al. 2017). Few studies to date, however, have described the use of specific yeast strains to guide the production of fortified *Daqu* or to improve the quality and flavour of the resultant liquor.

In present study, we isolated 16 yeast isolates from Chinese strong-flavour *Daqu* samples and subjected these isolates to RAPD analysis and identification. We further conducted a preliminary assessment of each yeast fermentation ability and its production of volatile compounds, and found strain YE006 (*S. cerevisiae*) exhibited the greatest ability to ferment ethanol but comparatively little ability to generate volatile compounds, whereas strain YE010 (*W. anomalus*) gave rise to the greatest quantity of volatile compounds despite its relatively poor fermentation efficiency. As such, we developed a process for producing superior fortified *Daqu* via the co-culture of YE006 and YE010. We additionally subjected the obtained fortified *Daqu* to traditional Chinese strong flavour liquor fermentation in order to efficiently improve the quality of the liquor. This study helps to provide a reliable approach to ensuring *Daqu* quality and improving the consistency and flavour of Chinese strong-flavour liquor through bioaugmentation.

## Materials and methods

### Sampling and yeast isolation

Blocks of fortified *Daqu* were obtained from Anhui Yingjia Distillery Group Co., Ltd. in LuAn city, Anhui Province, China. Samples of *Daqu* powder were then prepared by grinding, pooling, and sieving these samples through a 20-mesh to yield a more consistent sample. Samples of *Daqu* powder (10 g) were then combined with 90 mL of sterile physiological saline and serial tenfold dilutions thereof were prepared. Next, 100 μL aliquots were plated on yeast extract peptone dextrose medium (YEPD) (yeast extract 10 g, peptone 20 g, dextrose 20 g, agar 18 g, in 1 L of dH2O) plates supplemented with 0.1 g/L of chloramphenicol. Plates were incubated for 48 h at 30 °C under aerobic conditions, after which triplicate samples were counted to establish the number of colonies in each dilution. A total of 5–10 isolates were then selected from the three highest dilutions based upon colony morphology, and these isolates were purified via at least three successive rounds of streaking onto YEPD agar plates. Isolates were then grown for 24 h in YEPD broth at 30 °C, followed by storage at 4 °C for additional evaluation.

### Random amplified polymorphic DNA (RAPD) analysis

A DNA extraction kit (Sangon Biotech, Shanghai, China) was utilized to extract gDNA from yeast isolates, after which these isolated strains were classified via RAPD analysis using the M13 primer (5′GAGGAGGGTGGC GGTTCT 3′) (Andrighetto et al. 2000). PCR reactions were conducted in a 25 µL volume containing 2.5 µL of 10 × buffer (20 mM Mg^2+^ plus), 2 µL of dNTPs (2.5 mM), 1.5 µL of primers (10 µM), 2 µL of DNA template, 0.25 µL of ExTaq (5 U/µL), and 16.75 µL of ddH_2_O. Thermocycler settings were: 94 °C for 5 min; 34 cycles of 94 °C for 1 min, 45 °C for 2 min and 72 °C for 1.5 min; 72 °C for 10 min. The resultant PCR product was then assessed via 1% agarose gel electrophoresis.

Yeast identification was conducted using the universal NL1 and NL4 primer pair, which was used to sequence strains in each of the RAPD fingerprint groups (Yan et al. 2019). Sequences were then aligned with the GenBank database 26S rRNA gene sequences with the BLAST algorithm tool. Nucleotide sequences from this study were assigned GenBank Accession Nos. MW076944-MW076959 (Table 1).

**Table 1:** Isolated yeast strains identities following purification

No	GenBank accession number	Sequence similarity (%)	Closest relative
YE001	MW076944	100	*Hanseniaspora vineae*
YE002	MW076945	99	*Pichia kluyveri*
YE003	MW076946	100	*Trichosporon asahii*
YE004	MW076947	100	*Pichia kluyveri*
YE005	MW076948	100	*Hanseniaspora vineae*
YE006	MW076949	100	*Saccharomyces cerevisiae*
YE007	MW076950	100	*Wickerhamomyces anomalus*
YE008	MW076951	100	*Kluyveromyces lactis*
YE009	MW076952	100	*Saccharomyces cerevisiae*
YE010	MW076953	100	*Wickerhamomyces anomalus*
YE011	MW076954	100	*Yarrowia lipolytica*
YE012	MW076955	100	*Wickerhamomyces mori*
YE013	MW076956	100	*Galactomyces geotrichum*
YE014	MW076957	100	*Dabaryomyces hansenii*
YE015	MW076958	100	*Wickerhamomyces mori*
YE016	MW076959	100	*Saccharomyces kudriavzevii*

### Assessment of yeast fermentation activity

The ability of yeast isolates obtained from *Daqu* samples to facilitate ethanol fermentation was assessed as detailed previously by Liu et al. (2018). Briefly, individual colonies of each yeast isolate were grown for 24 h in liquid YEPD medium at 30℃ at 200 rpm. Next, these yeast cultures were transferred to a 250 mL Erlenmeyer flask containing 200 mL of YEPD medium and 190 g/L glucose at a 5% (v/v) ratio (approximately 1 × 10^7^ CFU/mL). Perforated 0.45 mm filter silicone stoppers (Merck Millipore, Italy) were affixed to these flasks to permit CO_2_ release while preventing contamination. Samples were prepared in triplicate and cultured under static conditions at 30 °C. Following fermentation, broth samples were collected to measure the remaining reducing sugar content and the amount of ethanol therein.

### Fortified *Daqu* preparation

**Fig. 1:**
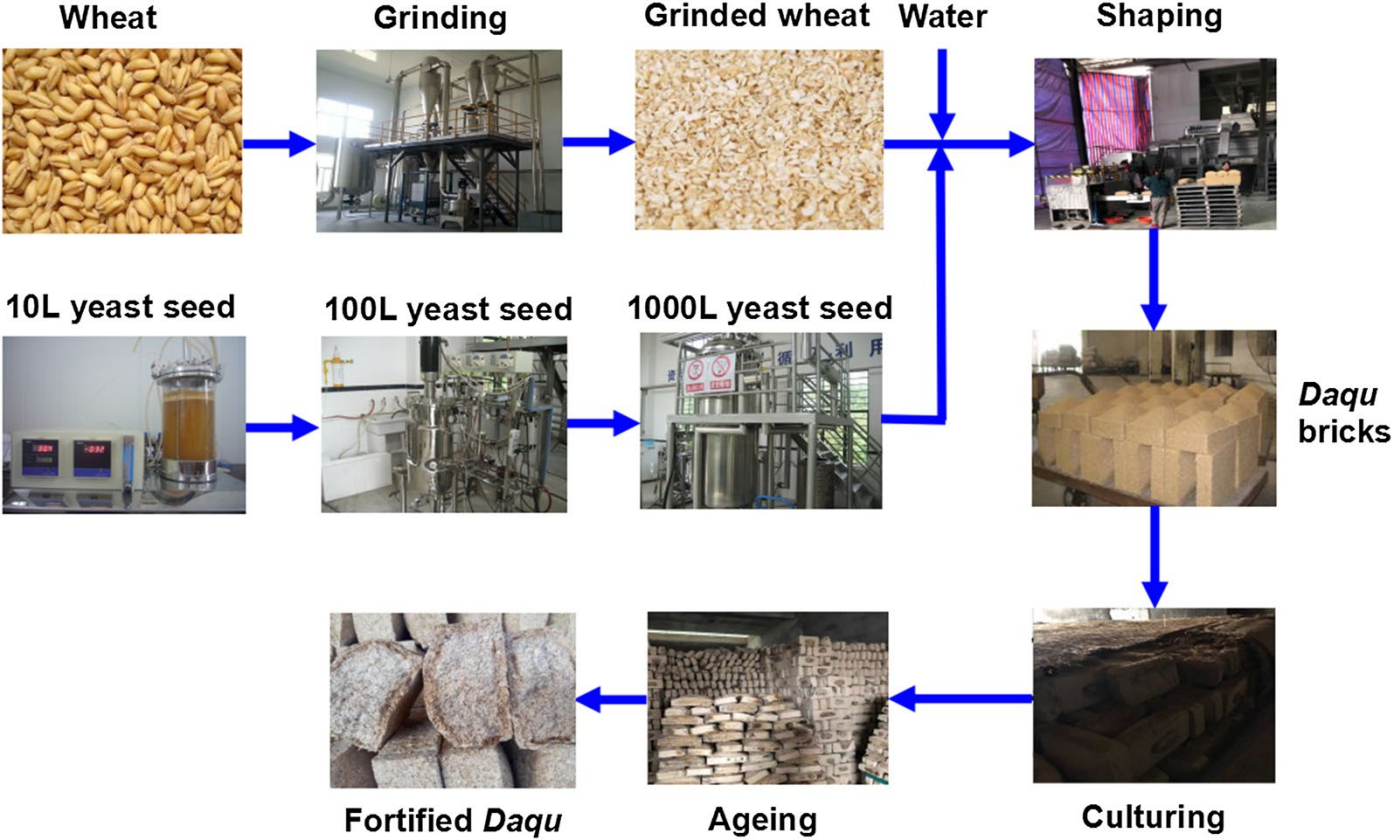
The principle of the process for fortified Chinese strong flavour *Daqu* production

Fortified *Daqu* was prepared as previously reported by Zheng et al. (2011) and as shown in Fig. 1. First, wheat was ground to release the starch in order to enhance water absorption. The ground wheat was then combined with water at a 38% (w/w) ratio, after which 1% (v/w) of each isolated yeast strain (10^7^ cells/mL) was added. The wet wheat mixture was then pressed to form a firm ~ 5.0 kg cube-shaped brick. These *Daqu* bricks were stacked in layers in a *Qu Fang* fermentation room for approximately 30 days during which humidity and temperature were carefully controlled through forced ventilation. The *Daqu* bricks were then transferred to another *Qu Fang* to age for an additional 3 months until mature.

To ensure sample representativeness, *Daqu* bricks from each layer were selected from random locations in triplicate. These triplicate samples were then ground, mixed together in a ~ 500 g sample, transferred to a sterile polyethylene bag, and stored at − 20 °C for subsequent analysis.

### Inoculum size optimization

In order to assess the impact of inoculum size on the production of fortified *Daqu,* a range of inoculum sizes (2%, 4%, and 6%) were tested, with these inoculums having been prepared from mixed cultures of YE006 and YE010 that had been grown for 24 h and mixed at a range of ratios (2:1, 1: 1, 1: 2, v/v). Inoculums were used to prepare fortified *Daqu* as detailed above. The sensory properties and volatile compounds associated with the resultant *Daqu* preparations were then assessed. All analyses were repeated in triplicate.

### Analytical methods

Standard *Daqu* analytical approaches were used for quantitative analyses of liquefying and saccharifying power for our prepared *Daqu* samples (Yan et al. 2019). One unit of liquefying and saccharifying power were respectively defined as the amount of starch liquefied and glucose liberated per hour by 1 g of *Daqu* in a sodium acetate buffer (50 mmol/L, pH 4.6) at 35 °C. National professional standard approaches were used to quantify esterifying and fermenting power (QB/T 4257-2011, 2011). Esterifying power was defined based upon ethyl caproate production by 50 g of *Daqu* over a 7-day period in a caproic acid and ethanol mixture at 35 °C. A unit of fermenting power was the amount of CO_2_ generated over a 72 h period by 1 g of *Daqu* at 30 °C. The Folin-Ciocalteu method was used to assess protease activity at 680 nm (Yan et al. 2019), with a unit of protease activity being defined as the quantity of tyrosine hydrolyzed from casein by 1 g of Daqu in 1 min under assay conditions. Analyses were performed in triplicate based upon sample dry weight.

### *Daqu* volatile compound analysis

Headspace solid-phase microextraction combined with gas chromatography-mass spectrometry (HS–SPME–GC–MS) was utilized to assess volatile compounds within *Daqu* preparations. Volatiles were extracted with a 50/30 μm DVB/CAR/ PDMS fiber (Supelco, PA, USA). Briefly, a 1.0 g sample of *Daqu* powder was combined in a 10 mL headspace vial with 10 μL of internal standard, after which this vial was equilibrated for 15 min 60 °C with constant stirring, followed by an additional 50 min incubation at 500 rpm.

After the extraction process was complete, SPME fibers were retracted into the needle and inserted into the GC–MS system injection port, followed by thermal desorption for 3 min at 250 °C. Compounds were then introduced into the analytical column. A Trace GC Ultra gas chromatograph-DSQ II mass spectrometer (Thermo Electron Corporation, MA, USA) and a TR-5 MS capillary column (30.0 m × 0.25 mm × 0.25 μm, J&W, CA, USA) were used to detect volatile compounds using settings published in a prior report (Yan et al. 2019). Volatile compounds were analyzed in triplicate as a function of dry weight.

### Sensory evaluation

The sensory properties of Chinese strong-flavour liquor samples were assessed by eight study participants (four males with over three years of experience in sensory experiments and four females with over three years of professional experience in the experimental evaluation of Chinese strong-flavour liquor). Prior to assessment, participants were familiarized with the criteria for sensory rating. The participants utilized a 10-point scale (1 = extremely dislike; 10 = extremely like) to rate the quality of fortified liquor sample properties including taste, flavour, mouthfeel, aftertaste, and overall acceptability (Yan et al. 2015).

## Results

### Preliminary screening of yeast strains

**Fig. 2:**
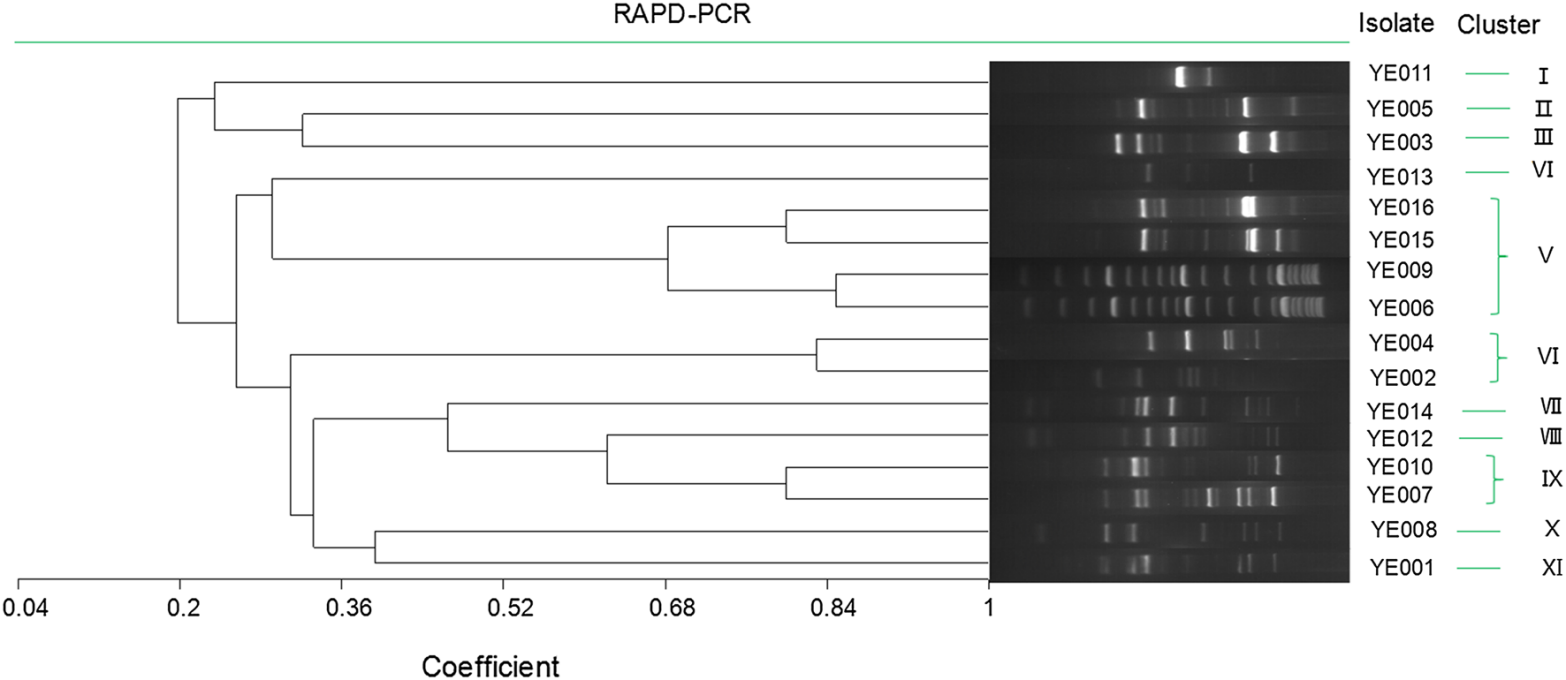
Cluster analysis of RAPD-PCR patterns obtained with primers M13

In total, 16 yeast colonies exhibiting different morphological characteristics were isolated from samples of Chinese strong-flavour *Daqu* and were evaluated via a RAPD-PCR approach which grouped these isolates into 11 clusters at a 68% similarity level (Fig. 2). Four of these yeast isolates (YE006, YE009, YE015, and YE016) were found in cluster V, while strains YE002 and YE004 were incorporated into group VI and strains YE007 and YE010 were incorporated into cluster IX. The remaining isolates (YE001, YE003, YE005, YE008, and YE011) were each incorporated into individual clusters.

Yeast isolate ITS sequences were aligned to representative sequences in the GenBank database using the BLAST tool, and were assigned the accession numbers MW076944-MW076959 (Table 1). At a similarity level of > 99%, these yeast isolates were identified as *Hanseniaspora vineae* (YE001, YE005), *Pichia kluyveri* (YE002, YE004), *Trichosporon asahii* (YE003), *Saccharomyces cerevisiae* (YE006, YE009), *Kluyveromyces lactis* (YE008), *Wickerhamomyces anomalus* (YE007, YE010)*, Yarrowia lipolytica* (YE011), *Wickerhamomyces mori* (YE012, YE015), *Galactomyces geotrichum* (YE013), *Dabaryomyces hansenii* (YE014), and *Saccharomyces kudriavzevii* (YE016) (Table 1). Of these, *Wickerhamomyces mori*, *Hanseniaspora vineae, Saccharomyces cerevisiae*, *Pichia kluyveri*, *Yarrowia lipolytica* were previously shown to be commonly found within Chinese strong-flavour *Daqu* sample ecosystems (Yan and Tong 2019; Xiang et al. 2020), although two other species reported in these prior studies (*Saccharomycopsis fibuligera* and *Issatchenkia orientalis*) were not detected in the present study. These species were, however, detected in another prior study assessing the microbial ecosystem within *Daqu* samples from Luzhou Laojiao liquor breweries in Sichuan Province, China (Zhang et al. 2014a, b).

### Assessment of yeast fermentation ability

**Fig. 3:**
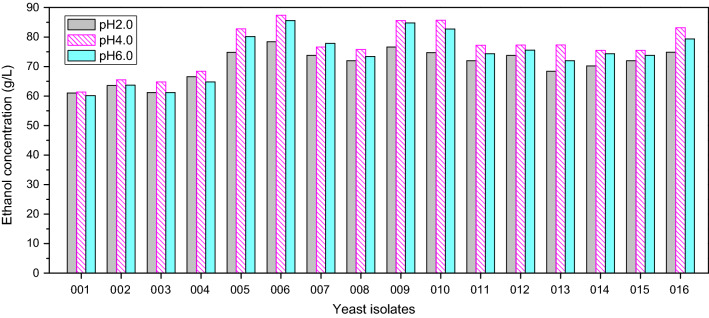
Profile of ethanol production by different yeasts fermenting glucose medium with three different initial pH

We next tested the fermentation abilities of our 16 yeast isolates (Fig. 3) by adding them to a synthetic medium solution containing glucose (180 g/L) at a starting pH of 2.0, 4.0, or 6.0 for 4 days. These pH levels were selected owing to the variability in the *Zaopei* of Chinese strong-flavour liquor depending on the industrial context in which it is prepared with the goal of assessing the pH dependence of any fermentation activity. We found that six isolates mediated significant reductions in sugar levels and high ethanol yields independent of starting pH, including YE005 (*Hanseniaspora vineae*), YE006 (*Saccharomyces cerevisiae*), YE009 (*Saccharomyces cerevisiae*), YE010 (*Wickerhamomyces anomalus*)*,* and YE016 (*Saccharomyces kudriavzevii*) (Fig. 3). The YE006 strain (*Saccharomyces cerevisiae*) exhibited the best fermentation activity, yielding values of 79.4 g/L, 88.4 g/L, and 86.6 g/L at starting pH values of 2.0, 4.0, and 6.0, respectively.

We next tested the ability of these 16 different yeast isolates to ferment sugars derived from wheat, which is the primary raw material used in the production of these *Daqu* samples. The four primary sugar types found in wheat flour include maltose, glucose, sucrose, and fructose. We therefore separately investigated the ability of our yeast isolates to ferment these four individual carbohydrates. All strains were able to ferment glucose and fructose (Additional file 1: Table S1) to yield acid and gas production, with the exception of strains YE003 (*Hanseniaspora vineae*) and YE013 (*Hanseniaspora vineae*), which failed to generate any gas. Significantly more variability was observed with respect to the fermentation of the disaccharides sucrose and maltose. Strains YE002 (*Pichia kluyveri*) and YE004 (*Pichia kluyveri*) were unable to produce gas via the fermentation of these sugars. Observed fermentation profiles were consistent with a prior study (Liu et al. 2018). Strains YE008 (*Kluyveromyces lactis*) and YE013 (*Galactomyces geotrichum*) exhibited a relatively limited ability to ferment fructose, whereas strains YE001 (*Hanseniaspora vineae*) and YE005 (*Hanseniaspora vineae*) were able to ferment glucose, sucrose, and fructose yet were largely unable to efficiently ferment maltose, which was the most common sugar found in wheat flour (Additional file 1: Table S1).

### Assessment of volatile compounds in fortified *Daqu* samples fermented with different yeasts

SPME-GC/MS is an effective means of detecting microorganism-derived volatile compounds (Liu et al. 2018), and has been used to analyze the flavour profiles of foods including *Daqu* (Yan et al. 2019). Herein, we therefore used this approach to assess the volatile compounds in Chinese fortified *Daqu* fermented using these different yeast strains. In total, we identified and classified 74 different volatile compounds (Additional file 1: Table S2), including esters (17), acids (15), alcohols (14), alkenes (9), aldehydes (8), volatile phenols (3), pyrazines (3), ketones (3), pyrrole (1), and furan (1). Of these compounds, esters were the most prevalent, followed by alcohols, acids, alkenes, ketones, aldehydes, and small amounts of volatile phenols, pyrroles, and furans.

There were significant differences in the concentrations of these different volatile compounds depending on the yeast strains used for fermentation (Additional file 1: Table S2). Control samples exhibited higher levels of volatile phenols (4-vinylphenol (VP1), 4-vinyl guaiacol (VP2), and 2-methoxy-4-vinylphenol (VP3)), presumably originating from *p*-coumaric and ferulic acids via enzymatic or thermal decarboxylation (Ye et al., 2014). In contrast, linoleic acid (AC13), oleic acid (AC14), ethyl acetate (ES1), ethyl isobutanoat (ES2), ethyl butanoate (ES3), 3-ethoxy-1-propanol (AL1), 1-hexanol (AL4), 2-methyl-1-propanol (AL5), 1-octen-3-ol (AL6), enanthol (AL7), iooctanol (AL8), octanol (AL10), 1-nonanol (AL11), 2-heptenal (AD1), nonaldehyde (AD2), 3-methyl-pentanoic acid (AC5), 2-methyl-butanoic acid (AC6), 2-nonanone (KE2), 3-hydroxy-2-butanone(Acetoin) (KE3), and tetramethylethylene (AK1) are likely derived from yeasts, as they were not detected in control samples.

**Fig. 4:**
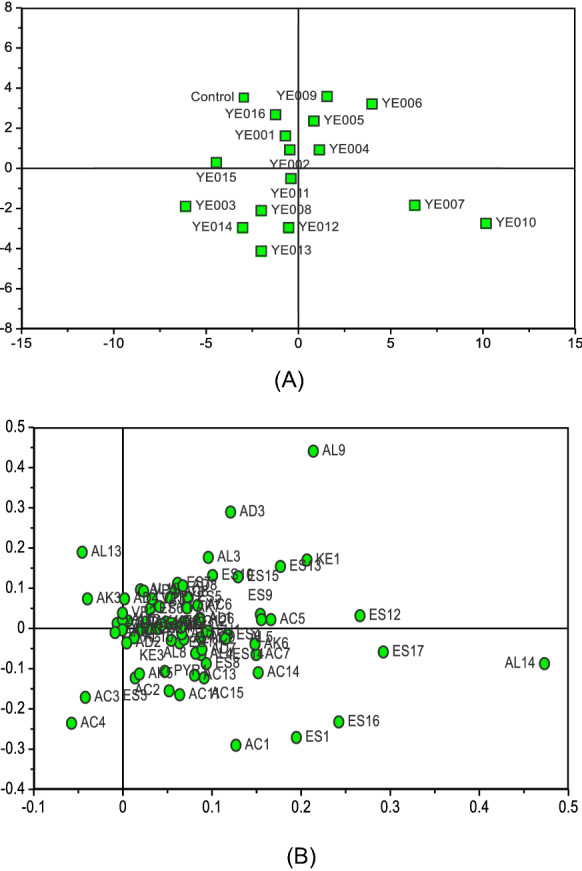
The PLS-DA score plot (**A**) and loading plot (**B**) showing the influence of yeast species on the volatile compounds of Chinese strong flavour *Daqu*. The first two principal components explained 60% of the total variance. YE001, YE002, YE003, YE004, YE005, YE006, YE007, YE008, YE009, YE010, YE011, YE012, YE013, YE014, YE015, YE016 refer to the *Daqu* fermented with yeast strain YE001, YE002, YE003, YE004, YE005, YE006, YE007, YE008, YE009, YE010, YE011, YE012, YE013, YE014, YE015, YE016, respectively. The control refers to the traditional production of Chinese strong flavour *Daqu*

The first two components of a PLS-DA effectively separated samples of fortified *Daqu* fermented using our different yeast isolates (Fig. 4A), highlighting differences in volatile compound production. *Daqu* prepared using stain YE010 (*Wickerhamomyces anomalus*) exhibited relatively high acetic acid (AC1), propionic acid (AC2), 3-methyl-pentanoic acid (AC5), 2-methyl-butanoic acid (AC6), octanol acid (AC7), nonanoic acid (AC8), benzoic acid (AC9), palmitic acid (AC11), linoleic acid (AC13), oleic acid (AC14), 9-hexadecenoic acid (AC15), ethyl acetate (ES1), hexanoic acid ethyl ester (ES4), undecanoic acid ethyl ester (ES6), nonanoic acid ethyl ester (ES7), ethyl decanoate (ES8), benzeneacetic acid ethyl ester (ES9), ethyl laurate (ES10), γ-nonylactone (ES11), ethyl oleate (ES12), tetradecanoic acid ethyl ester (ES13), ethyl pentadecanoate (ES14), ethyl 9-hexadecenoate (ES15), ethyl palmitate (ES16), ethyl linoleate (ES17), 3-ethoxy-1-propanol (AL1), 1-hexanol (AL4), 2-methyl-1-propanol (AL5), iooctanol (AL8), 1-nonanol (AL11), phenylethyl alcohol (AL14), 2-heptenal (AD1), pentanal (AD6), 2-phenyl-2-butenal (AD7), 3-hydroxy-2-butanone(Acetoin) (KE3), decamethylcyclopentasiloxane (AK2), tetradecane (AK4), pentadecane (AK5), caryophyllene (AK6), hexadecane (AK7), octamethylcyclotetrasiloxane (AK9), 2-methylpyrazine (PY1), 2,6-dimethylpyrazine (PY2), 2,3,5-trimethylpyrazine (PY3), 2-acetylpyrrole (PYR1), and 2-hexanoylfuran (FU1) levels (Fig. 4B and Additional file 1: Table S2). Many of these compounds have previously been reported to be common in Chinese strong-flavour *Daqu* (Yan et al. 2019). These samples contained the highest levels of phenylethyl alcohol (Additional file 1: Table S2), which is an alcohol generated via the Ehrlich pathway, which transforms branched-chain amino acids into alcohols (Hazelwood et al. 2008). Phenylethyl alcohol is a phenylalanine derivative that exhibits a rose-honey-like odor, which is associated with the aroma of final *Daqu* preparations (Pico et al. 2015). Similarly, we observed the presence of the fusel alcohols 2-methyl-1-propanol, 3-methyl-1-butanol, 2,3-butanediol, isoamyl alcohol, and octanol, which can be produced by yeasts from sugars and selected amino acids (typically branched-chain and aromatic amino acids) via the anabolic and Ehrlich pathways, respectively. Of these, 2,3-butanediol is commonly cited in studies of Chinese fortified liquor flavor, and is closely associated with the aroma of the resultant liquor, imparting it with a “sweet, mellow, alcoholic” flavor (Yan et al. 2015).

We found that samples of fortified *Daqu* fermented by *Saccharomyces cerevisiae* exhibited relatively high levels of isoamyl alcohol (AL3), 2,3-butanediol (AL9), 2-furanmethanol (AL12), benzyl alcohol (AL13), 2-heptenal (AD1), benzaldehyde (AD3), benzeneacetaldehyde (AD4), 2-octanone (KE1), 2-nonanone (KE2), 3-hydroxy-2-butanone (Acetoin) (KE3), 2-methylpyrazine (PY1), 2,6-dimethylpyrazine (PY2), 2,3,5-trimethylpyrazine (PY3) (Fig. 4 and Additional file 1: Table S2). These compounds were also present in samples of fortified *Daqu* prepared with other yeast strains. Higher levels of 2-methylpropanoic acid and 3-methylbutanoic acid were detected in samples of fortified *Daqu* fermented with *Wickerhamomyces anomalus* (YE007 and YE010), potentially because large quantities of intermediate fusel aldehydes were oxidized to yield corresponding acids rather than undergoing reduction via the Ehrlich pathway to yield alcohols (Hazelwood et al. 2008). Ehrlich pathway-derived alcohols, aldehydes, and acids are key contributors to overall fortified *Daqu* flavour and aroma (Pico et al. 2015), potentially explaining the differential impact of individual yeast isolates on *Daqu* quality. Fortified *Daqu* fermented by *Hanseniaspora vineae* (YE002 and YE004) accumulated the largest amounts of ethyl isobutanoat (ES2), 2-undecenal (AD5), and 2-pyrrolecarbaldehyde (AD8) (Additional file 1: Table S2). Fortified *Daqu* prepared using *Yarrowia lipolytica* (YE011) exhibited highest concentrations of butyric acid (AC3), 3-methyl-pentanoic acid (AC5), tetradecanoic acid (AC10), undecanoic acid ethyl ester (ES6), nonaldehyde (AD2), benzeneacetaldehyde (AD4), 2-pyrrolecarbaldehyde (AD8), and decamethylcyclopentasiloxane (AK2), while samples produced with *Hanseniaspora vineae* (YE001, YE005) contained the highest levels of alkanes (Tetramethylethylene (AK1), dodecamethylcyclohexasiloxane (AK3), caryophyllene (AK6), hexadecane (AK7), octamethylcyclotetrasiloxane (AK9)) (Additional file 1: Table S2), which were also present in traditional *Daqu* fermentation (control) samples, with the exception of tetramethylethylene (AK1).

Additional file 1: Table S2 also revealed that *Daqu* samples fermented using strain YE010 exhibited the highest total concentrations of volatile acids (22.17 μg/mL), esters (103.382 μg/mL), alcohols (41.280 μg/mL), ketones (11.168 μg/mL), alkanes (1.502 μg/mL), and furans (2.995 μg/mL), while also containing relatively high levels of aldehydes (7.822 μg/mL), volatile phenols (1.716 μg/mL), and pyrrole (1.824 μg/mL). Fortified *Daqu* samples fermented using strain HN006 exhibited the highest levels of total aldehydes (8.847 μg/mL) and ketones (9.991 μg/mL), but contained relatively low levels of total volatile compounds compared to preparations made using other yeast strains.

Based upon these results, we concluded that yeast strain YE010 produced the greatest quantities of volatile compounds but exhibited only intermediate ability to produce ethanol via fermentation, whereas the *Saccharomyces* strain YE006 exhibited maximal ethanol production but a relatively poor flavor profile. As such, we selected these two strains for use in a mixed inoculum in an effort to produce higher quality fortified *Daqu*. Each strain of YE006 (*S. cerevisiae*) and YE010 (*W. anomalus*) was deposited in China Center of Industrial Culture Collection (CICC, Beijing, China) and assigned with a number of CICC 33495 and CICC 31091, respectively.

### Effect of inoculation on fortified *Daqu* volatile compound levels

We next evaluate the relationship between the strain YE006 and strain YE010 inoculum size and volatile compound levels in fortified *Daqu* samples (Additional file 1: Table S3). The levels of these volatile compounds varied significantly as a function of inoculum size, with marked increases in total volatile compound levels relative to the levels observed in control (unfortified) *Daqu.* Levels of volatile acids, esters, alcohols, aldehydes, ketones, alkenes, pyrazines, pyrrole, and furan were elevated in fortified *Daqu* relative to unfortified control samples, whereas volatile phenol levels fell slightly following inoculation. Maximal total volatile compound levels were achieved in fortified Daqu preparations prepared using a 4% (v/v) inoculum composed of YE006 and YE010 at a 1:2 (v/v) inoculation ratio.

**Fig. 5:**
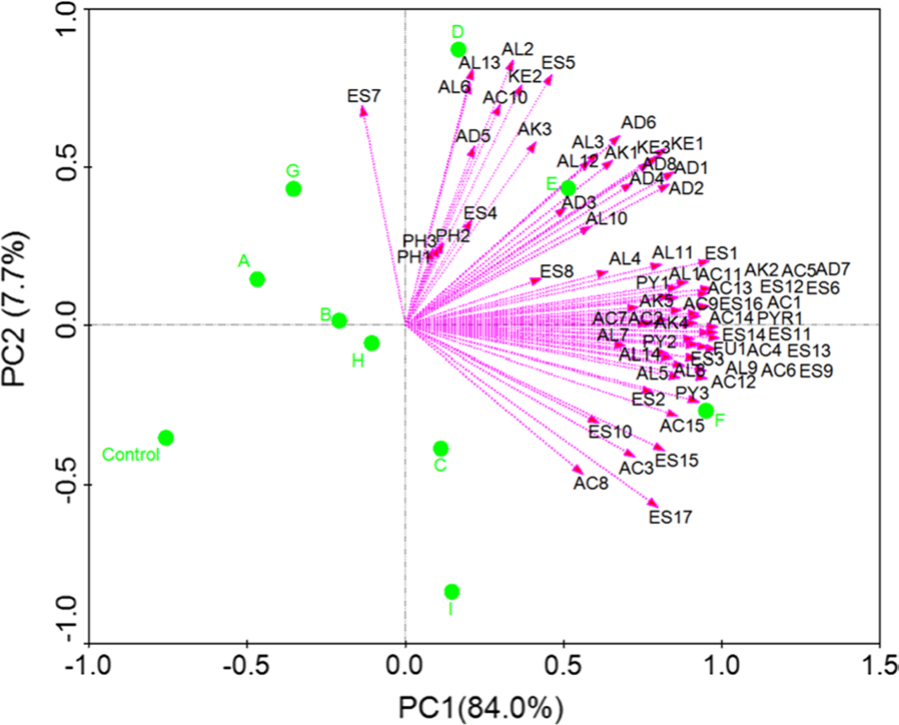
Principal component analysis (PCA) illustrating differences in volatile compounds from *Daqu* samples inoculated with different inoculation ratios of strains YE006 and the strain YE010. A: the inoculum size of 2% and YE006/YE010 inoculation ratio of 2: 1 (v/v); B: the inoculum size of 2% and YE006/YE010 inoculation ratio of 1: 1 (v/v); C: the inoculum size of 2% and YE006/YE010 inoculation ratio of 1: 2 (v/v); D: the inoculum size of 4% and YE006/YE010 inoculation ratio of 2: 1 (v/v); E: the inoculum size of 4% and YE006/YE010 inoculation ratio of 1: 1 (v/v); F: the inoculum size of 4% and YE006/YE010 inoculation ratio of 1: 2 (v/v); G: the inoculum size of 6% and YE006/YE010 inoculation ratio of 2: 1 (v/v); H: the inoculum size of 6% and YE006/YE010 inoculation ratio of 1: 1 (v/v); I: the inoculum size of 6% and YE006/YE010 inoculation ratio of 1: 2 (v/v); Control: traditional production of *Daqu*

To more fully assess the impact of bioaugmentation inoculation on volatile compound concentrations, a principal component analysis (PCA) was conducted to gauge the relationship between inoculum size and volatile compound content. The first principal component (PC1) accounted for 84.0% of observed variance, while PC2 accounted for 7.7% of this variance, with these two components thus accounting for 91.7% of overall variance consistent with good result representation (Fig. 5).

A PCA evaluation revealed the volatile compounds that were most important for the explanation of sample variability (Fig. 5). The AC1, AC2, AC3, AC4, AC5, AC6, AC7, AC8, AC9, AC11, AC12, AC13, AC14, AC15, AD7, AL1, AL4, AL7, AL11, AL14, AL8, AL9, AK2, AK4, AK5, AS6, ES1, ES2, ES3, ES8, ES9, ES10, ES11, ES12, ES13, ES14, ES15, ES16, ES17, PU1, PY2, PY3, and PYR1 volatile compounds are shown on the right side of the plot, and were strongly positively correlated with PC1 (0.9 or greater), indicating that the levels of these volatile compounds were significantly higher in samples of fortified *Daqu* inoculated with a 4% (v/v) inoculum composed of YE006 to YE010 at a 1: 2 (v/v) inoculation ratio (F), as shown in Additional file 1: Table S3.

### The impact of bioaugmentation on *Daqu* enzyme activity

**Fig. 6:**
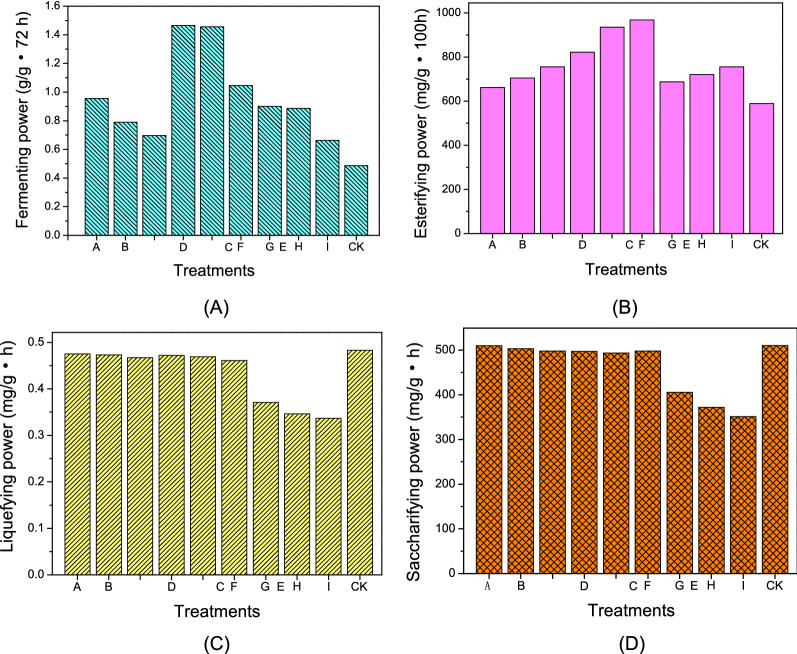
Effect of various bioaugmentation inoculation on the enzyme activity of *Daqu.* A: the inoculum size of 2% and YE006/YE010 inoculation ratio of 2: 1 (v/v); B: the inoculum size of 2% and YE006/YE010 inoculation ratio of 1: 1 (v/v); C: the inoculum size of 2% and YE006/YE010 inoculation ratio of 1: 2 (v/v); D: the inoculum size of 4% and YE006/YE010 inoculation ratio of 2: 1 (v/v); E: the inoculum size of 4% and YE006/YE010 inoculation ratio of 1: 1 (v/v); F: the inoculum size of 4% and YE006/YE010 inoculation ratio of 1: 2 (v/v); G: the inoculum size of 6% and YE006/YE010 inoculation ratio of 2: 1 (v/v); H: the inoculum size of 6% and YE006/YE010 inoculation ratio of 1: 1 (v/v); I: the inoculum size of 6% and YE006/YE010 inoculation ratio of 1: 2 (v/v); CK: traditional production of *Daqu*

Fermenting power is a key facet of *Daqu* that determines its utility for the production of Chinese strong-flavour liquor. Herein, we found that *Daqu* fermenting power rose with increasing inoculum size and with the relative levels of strain YE006 in mixed inoculum preparations, reaching maximal levels of 1.465 g CO_2_/g *Daqu***·**72 h when using a 2% inoculum composed of YE006/YE010 at a 1: 2 (v/v) inoculation ratio, with further increases in inoculum size or ratio having no beneficial impact on fermentation power (Fig. 6A). No significant differences in fermentation power were observed between fortified *Daqu* prepared using 2% and 4% inoculum sizes at the above ratio, and all fortified *Daqu* preparations exhibited superior liquefying power relative to traditional control *Daqu* (Fig. 6A).

Esterase activity is a primary mediator of volatile ester content in *Daqu*, and we found that bioaugmentation markedly enhanced the esterifying power of these preparations from 589.3 mg ethyl caproate/g.100 h to 968.2 mg ethyl caproate/g.100 h (Fig. 6B). Greater esterification power coincided well with relatively higher *Wickerhamomyces anomalus* abundance in our fortified *Daqu* preparations (Fig. 6B), as this yeast is known to secrete esterases (Yan et al. 2019). Maximal esterifying power was observed for a 2% inoculum composed of YE006/YE010 at a 1:2 (v/v) inoculation ratio (Fig. 6B).

Most yeast strains cannot directly utilize starches, which must first be hydrolyzed by amylase and glucoamylase activity to yield monosaccharides. Herein, we observed no significant changes in liquefying power when comparing 2% and 4% inoculums across a range of YE006/YE010 inoculation ratios, whereas such power decreases rapidly at higher 6% inoculum sizes (Fig. 6C), potentially due to the inhibition of *Bacillus* and mold growth by the higher abundance of yeast cells, thereby reducing amylase and glucoamylase production. Saccharifying power trends were similar to those observed for liquefying power, as shown in Fig. 4D.

## Discussion

Yeast plays important roles during the fermentation of Chinese strong flavour liquor. In present study, eleven yeast species were detected in Chinese strong flavour *Daqu*, and they were identified as *Hanseniaspora vineae* (YE001, YE005), *Pichia kluyveri* (YE002, YE004), *Trichosporon asahii* (YE003), *Saccharomyces cerevisiae* (YE006, YE009), *Kluyveromyces lactis* (YE008), *Wickerhamomyces anomalus* (YE007, YE010)*, Yarrowia lipolytica* (YE011), *Wickerhamomyces mori* (YE012, YE015), *Galactomyces geotrichum* (YE013), *Dabaryomyces hansenii* (YE014), and *Saccharomyces kudriavzevii* (YE016) (Table 1). Most yeast species detected in the present study have previously been detected in a range of other fermented foods and beverages, particularly in the context of fermented alcoholic beverages (Hu et al., 2017). We have previously shown that non-*Saccharomyces* species are dominant throughout the Chinese strong-flavour *Daqu* fermentation process, with *Saccharomyces* only composing a minority of the microbes within *Daqu* samples owing to their relatively poor tolerance for temperatures above 50 °C. In certain cases, however, *Saccharomyces* species can become dominant and serve as key mediators of alcoholic fermentation during the production of liquor (Yan and Tong 2019). Such results are consistent with work demonstrating the importance of non-*Saccharomyces* species in the winemaking process as key determinants of final wine flavour (Takush and Osborne 2009). Relatively little research, however, has been performed to assess the functional importance of non-*Saccharomyces* yeast species in the preparation of Chinese liquor.

Relative to unfortified *Daqu,* a clear increase in total levels of alcohols including ethanol was observed when using fortified *Daqu* inoculated with a combination of YE006 (*Saccharomyces cerevisiae*) and YE010 (*Wickerhamomyces anomalus*), potentially due to the increased fermentation, saccharifying, and liquefying power associated with this fortified preparation (Fig. 5). Ethanol is a key contributor to the overall aroma of a solution, and it can undergo conversion into aldehydes, esters, and other aroma-related substances through the actions of specific yeast isolates (Li et al. 2020). By producing *Daqu* using aroma-producing yeast (YE010) and ethanol-producing yeast (YE006), we were able to improve our overall fermentation efficiency while increasing the levels of esters and alcohol in the resultant preparations, markedly increasing ethyl caproate content and thus improving the quality of the Chinese strong-flavour liquor.

**Fig. 7:**
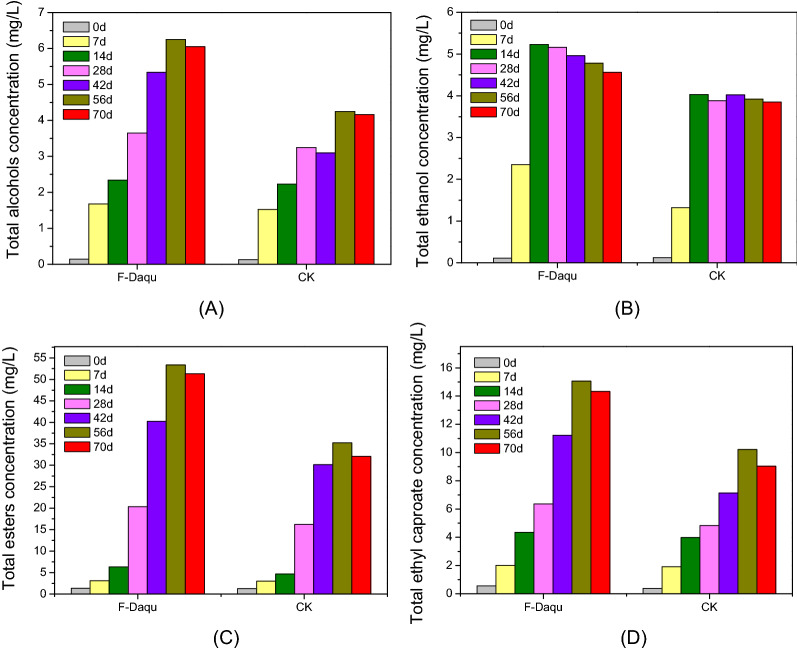
Concentration analysis of total alcohols, ethanol, esters, and ethyl caproate from different fermentation groups. **A** Total alcohols concentration; **B** Total ethanol concentration; **C** Total esters concentration; **D** Total ethyl caproate concentration. F-Daqu, Daqu fortified with co-culture of YE006 (*Saccharomyces cerevisiae*) and YE010 (*Wickerhamomyces anomalus*); CK: unfortified *Daqu*

The primary compounds that contribute to the overall flavour of Chinese strong-flavour liquor include alcohols, esters, organic acids, and other aromatic compounds. Esters are of particular importance in this context, and form in a yeast-strain-dependent manner such that different yeast isolates can impart different flavour profiles (Chen et al. 2014). Esters primarily contribute fruity flavors, with ethyl caproate contributing a sweet flavour that is characteristic of the overall flavour of Chinese strong-flavour liquor. We found that with increased fermentation time, total ester levels rose along with other volatile compounds in liquor preparations (Fig. 7). Total ester levels in liquor prepared with *Daqu* fortified with co-culture of YE006 (*Saccharomyces cerevisiae*) and YE010 (*Wickerhamomyces anomalus*) were increased relative to those in samples prepared with unfortified *Daqu* at later time points (d28 and d70). During the early and intermediate stages of the fermentation process (d0–d28), no significant differences were observed in ester levels among these preparations, with the advantages of fortified *Daqu* only manifesting during the more advanced stages of fermentation. Li et al. (2020) have previously provided two explanations for this phenomenon. For one, enzyme catalytic precursors only accumulate at significant levels during the later stages of fermentation. In addition, aroma-producing yeasts and other microbes in fortified *Daqu* only reach an optimal stable state at these later time points. The trends in ethyl caproate production in these samples were similar to overall trends in ester production (Fig. 7D).

**Fig. 8:**
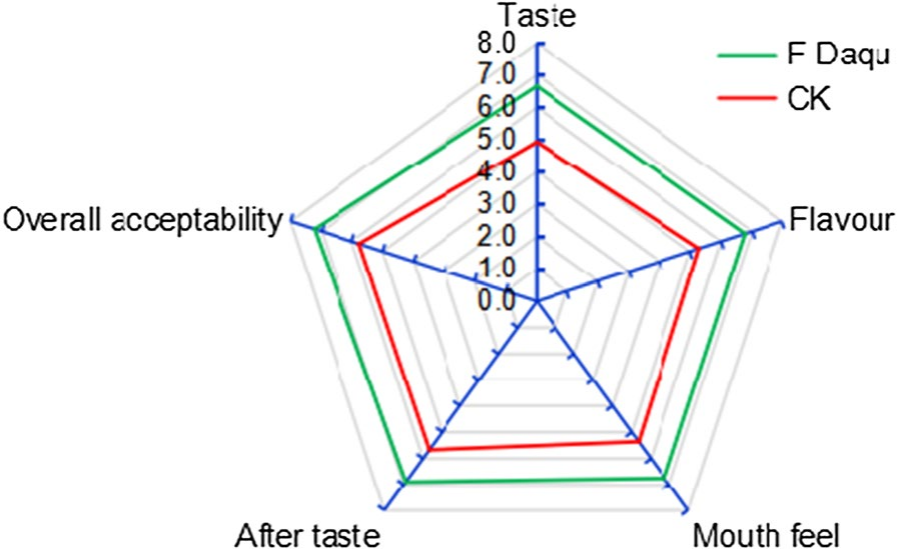
Sensory evaluation of obtained Chinese strong flavour liquor samples in different fermentations. F-Daqu: Daqu fortified with co-culture of YE006 (*Saccharomyces cerevisiae*) and YE010 (*Wickerhamomyces anomalus*); CK: unfortified *Daqu*

In addition, a sensory evaluation of the resultant liquor preparations was performed by experienced human study participants. These panelists scored liquors produced using our fortified *Daqu* higher with respect to taste, flavor, mouthfeel, aftertaste, and overall quality relative to liquor using a control unfortified *Daqu* (Fig. 8)*.* As such, fortified *Daqu* inoculated using a mixture of yeast strains YE006 and YE010 can be used to ferment Chinese strong-flavour liquor that exhibits better flavour and quality than liquor prepared using traditional unfortified *Daqu*. Yan et al. (2019) also found that co-fermentation using *S. cerevisiae* and *W. anomalus* was able to improve the fruity and floral profiles of rice wine. Together, these findings suggest that these two microorganisms can be reliably used in co-fermentation applications to yield consistent, high-quality Chinese strong-flavour liquors.

In total, we obtained 16 yeast isolates from Chinese strong-flavour *Daqu* samples and subjected these isolates to RAPD analysis and identification. We found that these yeast isolates varied in their relative abilities to ferment raw sugars and to produce the volatile compounds characteristic of fortified *Daqu.* Of these isolates, strain YE006 (*S. cerevisiae*) exhibited the greatest ability to ferment ethanol but comparatively little ability to generate volatile compounds, whereas strain YE010 (*W. anomalus*) gave rise to the greatest quantity of volatile compounds despite its relatively poor fermentation efficiency. As such, we combined these two yeast strains and used them in a mixed culture to generate superior fortified *Daqu,* developing optimized inoculation conditions that enabled us to maximize volatile compound production, fermentation power, esterifying power, saccharifying power, and liquefying power in resultant *Daqu* preparations. A 2% inoculum size at a 1:2 (v/v) inoculation ratio was ultimately found to maximize these properties and to improve the production of key flavour compounds in the resultant fortified liquor generated using this *Daqu,* which also attained higher sensory scores from study participants relative to that prepared using an unfortified control *Daqu* preparation. Overall, our findings may provide a reliable approach to ensuring *Daqu* quality and improving the consistency and flavour of Chinese strong-flavour liquor through bioaugmentation.

## Supplementary Information

**Additional file 1: Table S1.** Sugar fermentation profiles of different yeasts. **Table S2.** The volatile aroma compounds detected and measured in fortified *Daqu* obtained by using various yeast strains. **Table S3.** The volatile aroma compounds detected and measured in fortified *Daqu* obtained by using different inoculum sizes of strain YE006 and YE010.

## Data Availability

Please contact author for data requests.
