# Supplementary material for: Yeasts from Chinese strong flavour Daqu samples: isolation and evaluation of their potential for fortified Daqu production
Source: AMB Express. 2021 Dec 24;11:176. doi: 10.1186/s13568-021-01337-y (PMC8709808; doi:10.1186/s13568-021-01337-y)
Supplement: Supplementary file 1 — Additional file 1: Table S1. Sugar fermentation profiles of different yeasts. Table S2. The volatile aroma compounds detected and measured in fortified Daqu obtained by using various yeast strains. Table S3. The volatile aroma compounds detected and measured in fortified Daqu obtained by using different inoculum sizes of strain YE006 and YE010. [file 13568_2021_1337_MOESM1_ESM.doc]

**Supplementary materials**

**Table S1 Sugar fermentation profiles of different yeasts**

| Strain code | Glucose | Fructose | Maltose | Sucrose |
| --- | --- | --- | --- | --- |
| YE001 | + | + | w | + |
| YE002 | + | + | - | - |
| YE003 | + | - | + |  |
| YE004 | + | + | - | - |
| YE005 | + | + | w | + |
| YE006 | + | + | + | + |
| YE007 | + | + | + | + |
| YE008 | + | + | + | w |
| YE009 | + | + | + | + |
| YE010 | + | + | + | + |
| YE011 | + | + | + | + |
| YE012 | + | + | + | + |
| YE013 | + | - | + | w |
| YE014 | + | + | + | + |
| YE015 | + | + | + | + |
| YE016 | + | + | + | + |

+: positive −: negative w: weak positive.

Table S2 The volatile aroma compounds detected and measured in fortified *Daqu* obtained by using various yeast strains

| Number | Aroma compounds | Retention time (min) | Identification | Contents of volatile aroma compounds of 16 yeast strains/(μg/mL) | | | | | | | | | | | | | | | | |
| --- | --- | --- | --- | --- | --- | --- | --- | --- | --- | --- | --- | --- | --- | --- | --- | --- | --- | --- | --- | --- |
|  |  |  |  | YE001 | YE002 | YE003 | YE004 | YE005 | YE006 | YE007 | YE008 | YE009 | YE010 | YE011 | YE012 | YE013 | YE014 | YE015 | YE016 | Control |
|  | *Volatile acids* |  |  |  |  |  |  |  |  |  |  |  |  |  |  |  |  |  |  |  |
| AC1 | Acetic acid | 9.879 | MS, RI | 1.587 | 2.265 | 1.543 | 2.254 | 1.814 | 1.337 | 3.521 | 4.042 | 1.304 | 4.621 | 3.382 | 2.011 | 3.376 | 2.861 | 1.025 | 1.235 | 1.193 |
| AC2 | Propionic acid | 12.377 | MS, RI | 0.189 | 1.034 | 1.867 | 1.274 | 0.211 | 0.523 | 1.329 | 1.025 | 0.641 | 1.673 | 0.269 | 0.587 | 1.362 | 0.378 | 0.214 | 0.547 | 0.124 |
| AC3 | Butyric acid | 14.913 | MS, RI | 0.641 | 0.743 | 1.657 | 0.875 | 0.656 | 0.565 | 0.625 | 0.254 | 0.524 | 0.738 | 2.372 | 2.356 | 1.183 | 2.276 | ND | 0.642 | 0.421 |
| AC4 | Hexanoic acid | 16.214 | MS, RI | 0.481 | 0.623 | 2.456 | 0.647 | 0.501 | 0.557 | 0.867 | 1.798 | 0.425 | 0.875 | 1.601 | 1.257 | 2.487 | 1.057 | ND | 0.521 | 0.373 |
| AC5 | 3-methyl-pentanoic acid | 16.389 | MS, RI | 0.901 | 0.797 | 0.214 | 0.868 | 0.957 | 0.981 | 1.876 | ND | 0.921 | 2.014 | 1.014 | 0.574 | 0.741 | 0.247 | ND | 0.874 | 0.853 |
| AC6 | 2-methyl-butanoic acid | 15.588 | MS, RI | 0.358 | 0.421 | ND | 0.449 | 0.362 | 0.472 | 0.831 | ND | 0.402 | 0.842 | 0.454 | 0.128 | ND | 0.210 | 0.745 | 0.564 | 0.352 |
| AC7 | Octanol acid | 19.512 | MS, RI | 0.754 | 0.735 | 0.634 | 0.725 | 0.768 | 0.669 | 2.027 | 1.065 | 0.689 | 2.234 | 0.743 | 0.542 | 0.342 | 0.516 | 0.287 | 0.685 | 0.650 |
| AC8 | Nonanoic acid | 26.761 | MS, RI | 0.547 | 0.549 | ND | 0.621 | 0.574 | 0.612 | 0.602 | ND | 0.564 | 0.641 | 0.623 | 0.421 | 0.324 | ND | 0.854 | 0.367 | 0.545 |
| AC9 | Benzoic acid | 31.013 | MS, RI | 0.523 | 0.368 | 0.398 | 0.387 | 0.656 | 0.367 | 0.803 | 0.214 | 0.325 | 0.962 | 0.587 | 0.387 | 0.187 | 0.287 | 0.365 | 0.315 | 0.257 |
| AC10 | Tetradecanoic acid | 34.268 | MS, RI | 0.347 | 0.354 | 0.488 | 0.357 | 0.398 | 0.412 | 0.335 | 0.697 | 0.357 | 0.387 | 0.874 | 0.258 | ND | 0.397 | 0.412 | 0.367 | 0.321 |
| AC11 | Palmitic acid | 34.615 | MS, RI | 0.241 | 1.321 | 1.765 | 1.521 | 0.251 | 0.560 | 1.686 | 1.432 | 0.489 | 1.796 | 0.742 | 0.354 | 1.065 | 0.892 | 0.095 | 0.447 | 0.214 |
| AC12 | Pentadecanoic acid | 35.511 | MS, RI | 0.264 | 0.547 | ND | 0.745 | 0.275 | 0.492 | 0.671 | 0.325 | 0.421 | 0.721 | 0.632 | 0.741 | 0.025 | ND | 0.741 | 0.325 | 0.266 |
| AC13 | Linoleic acid | 36.047 | MS, RI | 0.117 | 0.258 | 0.125 | 0.354 | 0.201 | 0.402 | 0.856 | 0.328 | 0.354 | 1.047 | 0.103 | 1.214 | 0.685 | 0.137 | 0.541 | 0.395 | ND |
| AC14 | Oleic acid | 37.021 | MS, RI | 0.124 | 0.243 | 0.187 | 0.325 | 0.478 | 0.875 | 1.789 | 0.475 | 0.710 | 2.076 | 0.163 | 1.657 | 0.457 | 0.207 | 0.574 | 0.487 | ND |
| AC15 | 9-Hexadecenoic acid | 37.605 | MS, RI | 0.512 | 0.587 | 0.692 | 0.587 | 0.569 | 0.502 | 1.412 | 0.489 | 0.347 | 1.543 | 1.321 | 1.025 | 0.987 | 0.258 | 0.534 | 0.547 | 0.498 |
|  | Σ |  |  | 7.586 | 10.845 | 12.026 | 11.989 | 8.671 | 9.326 | 19.23 | 12.144 | 8.473 | 22.17 | 14.88 | 13.512 | 13.221 | 9.723 | 6.387 | 8.318 | 6.067 |
|  | *Esters* |  |  |  |  |  |  |  |  |  |  |  |  |  |  |  |  |  |  |  |
| ES1 | Ethyl acetate | 4.032 | MS, RI | 2.451 | 2.009 | 0.987 | 2.876 | 2.754 | 2.342 | 4.032 | 3.023 | 2.014 | 5.016 | 1.547 | 3.612 | 3.894 | 3.047 | 0.587 | 1.021 | ND |
| ES2 | Ethyl isobutanoat | 5.567 | MS, RI | 0.398 | 1.232 | 0.654 | 1.324 | 0.469 | 0.781 | 0.624 | 0.954 | 0.414 | 0.707 | 0.620 | 0.352 | 0.241 | 0.425 | 0.621 | 0.574 | ND |
| ES3 | Ethyl butanoate | 5.443 | MS, RI | 0.356 | 1.119 | 1.157 | 1.227 | 0.436 | 0.547 | 0.745 | 1.07 | 0.216 | 0.801 | 0.710 | 0.326 | 1.276 | 0.226 | 0.217 | 0.187 | ND |
| ES4 | Hexanoic acid ethyl ester | 6.049 | MS, RI | 2.723 | 2.258 | 2.089 | 2.574 | 2.758 | 2.826 | 3.178 | 2.587 | 2.174 | 3.854 | 2.874 | 2.541 | 2.587 | 2.057 | 2.987 | 2.867 | 2.682 |
| ES5 | Ethyl oenanthate | 8.239 | MS, RI | 0.667 | 0.597 | 0.687 | 0.821 | 0.621 | 1.176 | 0.634 | 0.357 | 0.724 | 0.874 | 0.657 | 0.541 | 0.369 | 0.287 | 0.321 | 0.574 | 0.543 |
| ES6 | Undecanoic acid ethyl ester | 10.661 | MS, RI | 2.868 | 3.047 | 3.987 | 3.568 | 2.897 | 2.974 | 3.023 | 3.657 | 2.887 | 3.574 | 3.856 | 1.697 | 2.065 | 1.876 | 2.874 | 2.697 | 2.850 |
| ES7 | Nonanoic acid ethyl ester | 12.976 | MS, RI | 2.865 | 3.287 | 2.321 | 3.379 | 3.975 | 2.981 | 3.075 | 2.934 | 2.774 | 3.874 | 3.367 | 1.987 | 2.174 | 3.641 | 3.254 | 2.747 | 2.704 |
| ES8 | Ethyl decanoate | 15.317 | MS, RI | 1.025 | 1.057 | 0.980 | 1.258 | 2.035 | 1.525 | 1.805 | 2.364 | 1.175 | 2.125 | 1.875 | 2.035 | 1.024 | 1.231 | 0.874 | 1.058 | 0.985 |
| ES9 | Benzeneacetic acid ethyl ester | 18.623 | MS, RI | 1.324 | 1.321 | 1.025 | 1.423 | 1.512 | 1.784 | 2.013 | 0.364 | 1.412 | 2.251 | 1.201 | 1.320 | 0.958 | 0.875 | 0.354 | 1.287 | 1.052 |
| ES10 | Ethyl laurate | 19.752 | MS, RI | 1.547 | 1.435 | 0.687 | 1.664 | 1.875 | 1.954 | 1.467 | 0.698 | 1.687 | 1.987 | 1.597 | 1.364 | 0.867 | 1.207 | 1.654 | 1.514 | 1.415 |
| ES11 | γ-nonylactone | 23.442 | MS, RI | 0.535 | 0.657 | 0.847 | 0.672 | 0.587 | 0.732 | 0.935 | 0.758 | 0.632 | 1.035 | 0.875 | 0.368 | 0.497 | 0.521 | 0.498 | 0.502 | 0.458 |
| ES12 | Ethyl oleate | 23.726 | MS, RI | 16.336 | 17.258 | 15.361 | 17.865 | 17.365 | 17.254 | 19.654 | 19.201 | 16.354 | 20.654 | 18.021 | 14.314 | 15.036 | 13.036 | 14.034 | 16.378 | 16.226 |
| ES13 | Tetradecanoic acid ethyl ester | 23.846 | MS, RI | 4.568 | 4.587 | 0.874 | 4.964 | 4.873 | 4.654 | 5.275 | 5.197 | 4.369 | 5.365 | 5.036 | 2.478 | 3.698 | 2.368 | 3.574 | 4.526 | 4.488 |
| ES14 | Ethyl pentadecanoate | 25.764 | MS, RI | 3.368 | 3.174 | 1.578 | 3.265 | 3.215 | 3.379 | 3.668 | 3.068 | 1.238 | 3.957 | 3.568 | 2.874 | 3.654 | 1.879 | 2.364 | 3.547 | 3.085 |
| ES15 | Ethyl 9-hexadecenoate | 28.161 | MS, RI | 3.355 | 3.424 | 3.021 | 3.514 | 3.479 | 3.654 | 4.045 | 2.875 | 3.025 | 4.125 | 3.621 | 2.874 | 2.036 | 1.698 | 2.874 | 3.684 | 3.331 |
| ES16 | Ethyl palmitate | 28.268 | MS, RI | 19.697 | 20.745 | 18.364 | 21.397 | 18.987 | 23.369 | 23.267 | 21.218 | 20.62 | 24.251 | 19.254 | 22.021 | 20.154 | 23.021 | 20.254 | 18.054 | 17.254 |
| ES17 | Ethyl linoleate | 31.429 | MS, RI | 13.987 | 13.906 | 10.361 | 14.874 | 14.657 | 15.654 | 16.932 | 8.265 | 14.157 | 18.932 | 15.687 | 15.987 | 16.254 | 13.874 | 12.087 | 13.658 | 13.786 |
|  | Σ |  |  | 78.07 | 81.113 | 64.98 | 86.665 | 82.495 | 87.586 | 94.372 | 78.59 | 75.872 | 103.382 | 84.366 | 76.691 | 76.784 | 71.269 | 69.428 | 74.875 | 70.859 |
|  | *Alcohols* |  |  |  |  |  |  |  |  |  |  |  |  |  |  |  |  |  |  |  |
| AL1 | 3-ethoxy-1-propanol | 1.358 | MS, RI | 0.362 | 0.276 | 0.478 | 0.368 | 0.875 | 0.734 | 0.845 | 0.387 | 0.653 | 0.976 | 0.256 | 0.369 | 0.321 | 0.178 | 0.521 | 0.856 | ND |
| AL2 | 3-methyl-butanol | 5.583 | MS, RI | 0.861 | 0.781 | 0.697 | 0.568 | 0.867 | 0.597 | 0.521 | 0.452 | 0.632 | 0.574 | 0.698 | 0.721 | 0.651 | 0.479 | 0.365 | 0.547 | 0.491 |
| AL3 | Isoamyl alcohol | 5.842 | MS, RI | 0.587 | 0.736 | 0.774 | 1.035 | 1.035 | 3.779 | 0.734 | 0.745 | 3.570 | 0.879 | 0.437 | 0.512 | 0.602 | 0.368 | 0.245 | 0.368 | 0.129 |
| AL4 | 1-hexanol | 7.281 | MS, RI | 0.214 | 0.256 | 0.198 | 0.325 | 0.452 | 0.923 | 0.875 | 0.889 | 0.294 | 0.982 | 0.314 | 0.542 | 0.218 | 0.533 | 0.546 | 0.332 | ND |
| AL5 | 2-methyl-1-propanol | 10.321 | MS, RI | 0.254 | 0.545 | 0.576 | 0.658 | 0.532 | 1.865 | 1.576 | 0.875 | 1.842 | 1.868 | 0.365 | 0.574 | 0.247 | 0.295 | 0.357 | 0.369 | ND |
| AL6 | 1-octen-3-ol | 11.11 | MS, RI | 0.697 | 0.382 | 0.366 | 0.421 | 0.632 | 0.553 | 0.401 | 0.402 | 0.432 | 0.421 | 0.588 | 0.463 | 0.467 | 0.598 | 0.652 | 0.424 | 0.312 |
| AL7 | Enanthol | 11.223 | MS, RI | 0.287 | 0.245 | 0.235 | 0.321 | 0.428 | 0.287 | 0.234 | 0.253 | 0.282 | 0.335 | 0.235 | 0.258 | 0.104 | 0.352 | 0.365 | 0.184 | ND |
| AL8 | Isooctanol | 12.107 | MS, RI | 0.362 | 0.267 | 0.221 | 0.362 | 0.412 | 0.443 | 0.482 | 0.432 | 0.376 | 0.887 | 0.456 | 0.625 | 0.222 | 0.412 | 0.254 | 0.365 | ND |
| AL9 | 2,3-butanediol | 13.399 | MS, RI | 4.231 | 4.565 | 3.985 | 4.874 | 4.658 | 5.965 | 4.032 | 4.201 | 5.687 | 4.235 | 3.698 | 3.875 | 3.178 | 4.161 | 3.368 | 4.452 | 4.675 |
| AL10 | Octanol | 13.611 | MS, RI | 0.321 | 0.234 | 0.195 | 0.469 | 0.487 | 0.517 | 0.254 | 0.218 | 0.445 | 0.323 | 0.257 | 0.321 | 0.563 | 0.745 | 0.241 | 0.412 | ND |
| AL11 | 1-nonanol | 15.966 | MS, RI | 0.202 | 0.138 | 0.123 | 0.321 | 0.312 | 0.376 | 0.431 | 0.210 | 0.230 | 0.503 | 0.352 | 0.321 | 0.187 | 0.214 | 0.264 | 0.320 | ND |
| AL12 | 2-Furanmethanol | 16.124 | MS, RI | 0.748 | 0.721 | 0.365 | 0.741 | 0.721 | 0.858 | 0.454 | 0.632 | 0.841 | 0.462 | 0.432 | 0.621 | 0.458 | 0.695 | 0.147 | 0.765 | 0.698 |
| AL13 | Benzyl alcohol | 20.635 | MS, RI | 0.656 | 1.423 | 0.956 | 1.345 | 0.787 | 1.956 | 0.305 | 0.365 | 1.458 | 0.367 | 0.745 | 0.636 | 0.654 | 1.035 | 1.654 | 1.847 | 1.234 |
| AL14 | Phenylethyl alcohol | 21.304 | MS, RI | 16.365 | 15.865 | 13.778 | 16.698 | 16.367 | 18.356 | 22.476 | 13.537 | 17.357 | 28.476 | 14.365 | 15.854 | 16.267 | 15.298 | 13.365 | 15.784 | 14.999 |
|  | Σ |  |  | 26.147 | 26.434 | 22.947 | 28.506 | 28.565 | 37.209 | 33.62 | 23.598 | 34.099 | 41.288 | 23.198 | 25.692 | 24.139 | 25.363 | 22.344 | 27.025 | 22.538 |
|  | *Aldehydes* |  |  |  |  |  |  |  |  |  |  |  |  |  |  |  |  |  |  |  |
| AD1 | 2-Heptenal | 7.882 | MS, RI | 0.32 | 0.254 | 0.167 | 0.321 | 0.541 | 0.879 | 0.754 | 0.188 | 0.763 | 0.652 | 0.231 | 0.547 | 0.385 | 0.741 | 0.687 | 0.215 | ND |
| AD2 | Nonaldehyde | 9.445 | MS, RI | 0.162 | 0.176 | 0.123 | 0.241 | 0.302 | 0.393 | 0.269 | 0.158 | 0.269 | 0.227 | 0.752 | 0.241 | 0.543 | 0.325 | 0.431 | 0.265 | ND |
| AD3 | Benzaldehyde | 12.705 | MS, RI | 1.254 | 2.126 | 0.343 | 2.365 | 1.412 | 2.798 | 1.646 | 0.227 | 2.676 | 1.596 | 0.148 | 0.365 | 0.136 | 0.117 | 0.512 | 2.158 | 1.950 |
| AD4 | Benzeneacetaldehyde | 15.487 | MS, RI | 0.568 | 0.321 | 0.214 | 0.365 | 0.524 | 0.641 | 0.357 | 0.541 | 0.569 | 0.357 | 0.654 | 0.128 | 0.365 | 0.523 | 0.541 | 0.454 | 0.479 |
| AD5 | 2-undecenal | 17.696 | MS, RI | 0.213 | 0.857 | 0.025 | 0.934 | 0.120 | 0.232 | 0.088 | 0.038 | 0.195 | 0.088 | 0.457 | 0.141 | 0.632 | 0.214 | 0.157 | 0.163 | ND |
| AD6 | Pentanal | 18.153 | MS, RI | 0.325 | 0.334 | 0.265 | 0.254 | 0.373 | 0.687 | 0.458 | 0.235 | 0.676 | 0.698 | 0.231 | 0.357 | 0.157 | 0.241 | 0.147 | 0.289 | ND |
| AD7 | 2-phenyl-2-butenal | 21.581 | MS, RI | 1.652 | 1.436 | 2.034 | 1.567 | 1.697 | 1.985 | 2.178 | 0.698 | 1.874 | 2.897 | 1.657 | 2.310 | 1.698 | 1.654 | 2.130 | 1.625 | 1.582 |
| AD8 | 2-Pyrrolecarbaldehyde | 23.613 | MS, RI | 1.032 | 1.542 | 0.985 | 1.365 | 1.187 | 1.232 | 1.062 | 0.362 | 1.152 | 1.252 | 1.735 | 0.684 | 0.587 | 0.365 | 0.587 | 1.021 | 0.931 |
|  | Σ |  |  | 5.526 | 7.046 | 4.156 | 7.412 | 6.156 | 8.847 | 6.812 | 2.447 | 8.174 | 7.767 | 5.865 | 4.773 | 4.503 | 4.18 | 5.192 | 6.19 | 4.942 |
|  | *Ketones* |  |  |  |  |  |  |  |  |  |  |  |  |  |  |  |  |  |  |  |
| KE1 | 2-octanone | 6.962 | MS, RI | 5.65 | 3.807 | 3.412 | 4.201 | 5.98 | 8.867 | 6.321 | 8.187 | 9.108 | 6.897 | 4.25 | 6.035 | 2.36 | 5.01 | 3.687 | 4.625 | 3.566 |
| KE2 | 2-nonanone | 9.352 | MS, RI | 0.259 | 0.133 | 0.178 | 0.352 | 0.321 | 0.456 | 0.301 | 0.113 | 0.408 | 0.357 | 0.254 | 0.363 | 0.231 | 0.152 | 0.321 | 0.258 | ND |
| KE3 | 3-hydroxy-2-butanone (Acetoin) | 9.611 | MS, RI | 0.137 | 0.204 | 0.234 | 0.243 | 0.251 | 0.668 | 0.459 | 0.152 | 0.652 | 0.568 | 0.474 | 0.521 | 0.437 | 0.468 | 0.412 | 0.326 | ND |
|  | Σ |  |  | 6.046 | 4.144 | 3.824 | 4.796 | 6.552 | 9.991 | 7.081 | 8.452 | 10.168 | 7.822 | 4.978 | 6.919 | 3.028 | 5.63 | 4.42 | 5.209 | 3.566 |
|  | *Alkanes* |  |  |  |  |  |  |  |  |  |  |  |  |  |  |  |  |  |  |  |
| AK1 | Tetramethylethylene | 4.321 | MS, RI | 0.296 | 0.085 | 0.138 | 0.216 | 0.387 | 0.254 | 0.196 | 0.321 | 0.292 | 0.185 | 0.126 | 0.235 | 0.198 | 0.134 | 0.136 | 0.235 | ND |
| AK2 | Decamethylcyclopentasiloxane | 4.781 | MS, RI | 0.621 | 0.125 | 0.624 | 0.538 | 0.657 | 0.516 | 0.603 | 0.354 | 0.465 | 0.832 | 0.487 | 0.463 | 0.254 | 0.536 | 0.456 | 0.533 | 0.441 |
| AK3 | Dodecamethylcyclohexasiloxane | 8.819 | MS, RI | 0.657 | 0.625 | 0.701 | 0.732 | 0.768 | 0.651 | 0.223 | 0.698 | 0.558 | 0.287 | 0.638 | 0.532 | 0.497 | 0.503 | 0.364 | 0.641 | 0.602 |
| AK4 | Tetradecane | 9.658 | MS, RI | 0.467 | 0.587 | 0.364 | 0.498 | 0.478 | 0.645 | 0.632 | 0.632 | 0.632 | 0.654 | 0.487 | 0.369 | 0.248 | 0.752 | 0.369 | 0.425 | 0.423 |
| AK5 | Pentadecane | 12.017 | MS, RI | 1.125 | 1.854 | 1.756 | 1.254 | 1.358 | 1.365 | 1.721 | 1.302 | 1.305 | 1.823 | 1.653 | 1.025 | 1.742 | 1.684 | 1.598 | 1.115 | 1.058 |
| AK6 | Caryophyllene | 14.099 | MS, RI | 5.365 | 4.021 | 3.654 | 3.981 | 5.036 | 3.871 | 5.054 | 3.026 | 3.541 | 5.143 | 5.038 | 4.185 | 4.369 | 5.031 | 2.364 | 3.647 | 2.495 |
| AK7 | Hexadecane | 14.333 | MS, RI | 0.992 | 0.587 | 0.697 | 0.628 | 1.178 | 0.929 | 0.986 | 0.258 | 0.918 | 1.082 | 0.856 | 0.368 | 0.854 | 0.952 | 0.254 | 0.875 | 0.804 |
| AK8 | α-Caryophyllene | 15.727 | MS, RI | 0.752 | 0.258 | 0.365 | 0.248 | 0.453 | 0.284 | 0.362 | 0.154 | 0.202 | 0.375 | 0.251 | 0.362 | 0.471 | 0.621 | 0.843 | 0.251 | 0.196 |
| AK9 | Octamethylcyclotetrasiloxane | 21.032 | MS, RI | 0.787 | 0.683 | 0.702 | 0.695 | 0.731 | 0.682 | 0.752 | 0.632 | 0.721 | 0.787 | 0.724 | 0.721 | 0.698 | 0.582 | 0.469 | 0.698 | 0.675 |
|  | Σ |  |  | 11.062 | 8.825 | 9.001 | 8.79 | 11.046 | 9.197 | 10.529 | 7.377 | 8.634 | 11.168 | 10.26 | 8.26 | 9.331 | 10.795 | 6.853 | 8.42 | 6.694 |
|  | *Volatile phenols* |  |  |  |  |  |  |  |  |  |  |  |  |  |  |  |  |  |  |  |
| VP1 | 4-Vinylphenol | 20.312 | MS, RI | 0.154 | 0.102 | 0.132 | 0.210 | 0.325 | 0.376 | 0.284 | 0.165 | 0.354 | 0.387 | 0.462 | 0.214 | 0.084 | 0.124 | 0.125 | 0.251 | 0.678 |
| VP2 | 4-Vinyl guaiacol | 23.345 | MS, RI | 0.231 | 0.245 | 0.158 | 0.258 | 0.251 | 0.214 | 0.223 | 0.196 | 0.203 | 0.318 | 0.417 | 0.322 | 0.136 | 0.172 | 0.362 | 0.208 | 0.568 |
| VP3 | 2-Methoxy-4-vinylphenol | 26.754 | MS, RI | 0.652 | 0.565 | 0.425 | 0.672 | 0.756 | 0.784 | 0.765 | 0.654 | 0.752 | 0.797 | 0.874 | 0.254 | 0.468 | 0.792 | 0.365 | 0.651 | 1.532 |
|  | Σ |  |  | 1.037 | 0.912 | 0.715 | 1.14 | 1.332 | 1.374 | 1.272 | 1.015 | 1.309 | 1.502 | 1.753 | 0.79 | 0.688 | 1.088 | 0.852 | 1.11 | 2.778 |
|  | *Pyrazine* |  |  |  |  |  |  |  |  |  |  |  |  |  |  |  |  |  |  |  |
| PY1 | 2-methylpyrazine | 6.769 | MS, RI | 0.251 | 0.321 | 0.251 | 0.361 | 0.267 | 0.514 | 0.365 | 0.175 | 0.585 | 0.395 | 0.298 | 0.254 | 0.360 | 0.247 | 0.268 | 0.478 | 0.240 |
| PY2 | 2,6-dimethylpyrazine | 8.132 | MS, RI | 0.312 | 0.332 | 0.154 | 0.368 | 0.274 | 0.568 | 0.281 | 0.263 | 0.514 | 0.395 | 0.301 | 0.142 | 0.172 | 0.214 | 0.168 | 0.310 | 0.241 |
| PY3 | 2,3,5-trimethylpyrazine | 9.797 | MS, RI | 0.656 | 0.585 | 0.435 | 0.610 | 0.668 | 0.721 | 0.620 | 0.568 | 0.787 | 0.926 | 0.634 | 0.674 | 0.703 | 0.539 | 0.621 | 0.598 | 0.446 |
|  | Σ |  |  | 1.219 | 1.238 | 0.84 | 1.339 | 1.209 | 1.803 | 1.266 | 1.006 | 1.886 | 1.716 | 1.233 | 1.07 | 1.235 | 1.00 | 1.057 | 1.386 | 0.927 |
|  | *Pyrrole* |  |  |  |  |  |  |  |  |  |  |  |  |  |  |  |  |  |  |  |
| PYR1 | 2-acetylpyrrole | 22.559 | MS, RI | 1.354 | 1.623 | 0.954 | 1.853 | 1.456 | 1.482 | 1.753 | 1.647 | 1.289 | 1.824 | 1.698 | 1.853 | 1.789 | 1.698 | 1.853 | 1.214 | 1.195 |
|  | Σ |  |  | 1.354 | 1.623 | 0.954 | 1.853 | 1.456 | 1.482 | 1.753 | 1.647 | 1.289 | 1.824 | 1.698 | 1.853 | 1.789 | 1.698 | 1.853 | 1.214 | 1.195 |
|  | *Furan* |  |  |  |  |  |  |  |  |  |  |  |  |  |  |  |  |  |  |  |
| FU1 | 2-Hexanoylfuran | 16.512 | MS, RI | 1.745 | 1.754 | 0.982 | 1.964 | 1.756 | 1.873 | 1.885 | 2.469 | 1.701 | 2.995 | 2.367 | 2.781 | 0.962 | 0.785 | 0.652 | 1.758 | 1.698 |
|  | Σ |  |  | 1.745 | 1.754 | 0.982 | 1.964 | 1.756 | 1.873 | 1.885 | 2.469 | 1.701 | 2.995 | 2.367 | 2.781 | 0.962 | 0.785 | 0.652 | 1.758 | 1.698 |

Table S3 The volatile aroma compounds detected and measured in fortified *Daqu* obtained by using different inoculum sizes of strain YE006 and YE010

| Number | Aroma compounds | Retention time (min) | Identification | Contents of volatile aroma compounds of fortified *Daqu* produced with different inoculum sizes and ratios of strain strain YE006 and strain YE010/(μg/mL) | | | | | | | | | |
| --- | --- | --- | --- | --- | --- | --- | --- | --- | --- | --- | --- | --- | --- |
|  |  |  |  | Control | 2% | | | 4% | | | 6% | | |
|  |  |  |  |  | A  (2: 1) | B  (1: 1) | C  (1: 2) | D  (2: 1) | E  (1: 1) | F  (1: 2) | G  (2: 1) | H  (1: 1) | I  (1: 2) |
|  | *Volatile acids* |  |  |  |  |  |  |  |  |  |  |  |  |
| AC1 | Acetic acid | 9.879 | MS, RI | 1.189 | 1.337 | 1.963 | 2.264 | 2.945 | 3.134 | 3.825 | 2.042 | 2.537 | 2.821 |
| AC2 | Propionic acid | 12.377 | MS, RI | 0.131 | 0.587 | 0.632 | 0.876 | 1.031 | 1.241 | 1.321 | 0.687 | 0.725 | 1.201 |
| AC3 | Butyric acid | 14.913 | MS, RI | 0.417 | 0.301 | 0.357 | 0.423 | 0.432 | 0.532 | 0.658 | 0.413 | 0.545 | 0.638 |
| AC4 | Hexanoic acid | 16.214 | MS, RI | 0.365 | 0.447 | 0.521 | 0.536 | 0.541 | 0.621 | 0.726 | 0.501 | 0.536 | 0.638 |
| AC5 | 3-methyl-Pentanoic acid | 16.389 | MS, RI | 0.847 | 0.967 | 1.326 | 1.468 | 1.654 | 1.898 | 2.012 | 1.456 | 1.568 | 1.865 |
| AC6 | 2-methyl-butanoic acid | 15.588 | MS, RI | 0.356 | 0.471 | 0.488 | 0.521 | 0.587 | 0.687 | 0.796 | 0.598 | 0.675 | 0.742 |
| AC7 | Octanol acid | 19.512 | MS, RI | 0.642 | 0.869 | 1.024 | 1.546 | 1.654 | 1.875 | 2.005 | 1.587 | 1.765 | 1.899 |
| AC8 | Nonanoic acid | 26.761 | MS, RI | 0.556 | 0.412 | 0.467 | 0.481 | 0.502 | 0.547 | 0.632 | 0.501 | 0.517 | 0.621 |
| AC9 | Benzoic acid | 31.013 | MS, RI | 0.236 | 0.467 | 0.598 | 0.698 | 0.765 | 0.825 | 0.875 | 0.614 | 0.767 | 0.832 |
| AC10 | Tetradecanoic acid | 34.268 | MS, RI | 0.317 | 0.432 | 0.378 | 0.328 | 0.458 | 0.417 | 0.404 | 0.417 | 0.402 | 0.387 |
| AC11 | Palmitic acid | 34.615 | MS, RI | 0.209 | 0.480 | 0.553 | 0.821 | 1.023 | 1.412 | 1.641 | 0.913 | 1.023 | 1.216 |
| AC12 | Pentadecanoic acid | 35.511 | MS, RI | 0.264 | 0.438 | 0.423 | 0.445 | 0.465 | 0.563 | 0.668 | 0.425 | 0.532 | 0.613 |
| AC13 | Linoleic acid | 36.047 | MS, RI | ND | 0.413 | 0.565 | 0.623 | 0.657 | 0.784 | 0.865 | 0.598 | 0.612 | 0.765 |
| AC14 | Oleic acid | 37.021 | MS, RI | ND | 0.721 | 0.992 | 1.225 | 1.278 | 1.654 | 1.954 | 0.954 | 1.247 | 1.745 |
| AC15 | 9-Hexadecenoic acid | 37.605 | MS, RI | 0.492 | 0.501 | 0.592 | 0.885 | 0.964 | 1.201 | 1.604 | 0.784 | 1.036 | 1.527 |
|  | Σ |  |  | 8.843 | 10.879 | 13.14 | 14.956 | 17.391 | 19.986 | 12.49 | 14.487 | 17.51 | 8.843 |
|  | *Esters* |  |  |  |  |  |  |  |  |  |  |  |  |
| ES1 | Ethyl acetate | 4.032 | MS, RI | ND | 1.042 | 1.954 | 2.873 | 3.655 | 4.335 | 5.041 | 2.436 | 2.645 | 2.818 |
| ES2 | Ethyl isobutanoat | 5.567 | MS, RI | ND | 0.483 | 0.524 | 0.612 | 0.434 | 0.573 | 0.768 | 0.404 | 0.526 | 0.723 |
| ES3 | Ethyl butanoate | 5.443 | MS, RI | ND | 0.201 | 0.261 | 0.319 | 0.321 | 0.446 | 0.536 | 0.264 | 0.396 | 0.501 |
| ES4 | Hexanoic acid, ethyl ester | 6.049 | MS, RI | 2.673 | 1.021 | 1.384 | 1.721 | 2.787 | 2.521 | 2.344 | 2.582 | 2.011 | 1.899 |
| ES5 | Ethyl oenanthate | 8.239 | MS, RI | 0.539 | 0.812 | 0.712 | 0.643 | 1.123 | 0.873 | 0.887 | 0.857 | 0.724 | 0.676 |
| ES6 | Undecanoic acid, ethyl ester | 10.661 | MS, RI | 1.842 | 1.965 | 2.283 | 2.728 | 2.865 | 3.174 | 3.610 | 2.054 | 2.587 | 2.945 |
| ES7 | Nonanoic acid ethyl ester | 12.976 | MS, RI | 2.697 | 2.885 | 2.452 | 1.862 | 3.554 | 2.887 | 2.025 | 2.243 | 1.632 | 1.982 |
| ES8 | Ethyl decanoate | 15.317 | MS, RI | 0.966 | 1.525 | 0.980 | 1.258 | 1.632 | 1.022 | 3.024 | 2.364 | 1.875 | 1.125 |
| ES9 | Benzeneacetic acid ethyl ester | 18.623 | MS, RI | 1.047 | 1.284 | 1.425 | 1.523 | 1.612 | 1.784 | 2.257 | 1.264 | 1.412 | 2.012 |
| ES10 | Ethyl laurate | 19.752 | MS, RI | 1.408 | 0.551 | 0.603 | 0.753 | 0.889 | 1.653 | 1.965 | 0.798 | 1.213 | 1.432 |
| ES11 | γ-nonylactone | 23.442 | MS, RI | 0.434 | 0.533 | 0.643 | 0.751 | 0.886 | 0.973 | 1.135 | 0.757 | 0.873 | 0.965 |
| ES12 | Ethyl oleate | 23.726 | MS, RI | 15.887 | 16.879 | 18.356 | 20.776 | 21.897 | 23.567 | 24.053 | 17.201 | 18.354 | 20.231 |
| ES13 | Tetradecanoic acid ethyl ester | 23.846 | MS, RI | 4.387 | 4.442 | 4.574 | 4.764 | 4.873 | 5.055 | 5.353 | 4.798 | 4.965 | 5.013 |
| ES14 | Ethyl pentadecanoate | 25.764 | MS, RI | 3.001 | 3.173 | 3.378 | 3.450 | 3.515 | 3.668 | 3.915 | 3.019 | 3.238 | 3.450 |
| ES15 | Ethyl 9-hexadecenoate | 28.161 | MS, RI | 3.322 | 3.354 | 3.401 | 3.414 | 3.479 | 3.654 | 4.104 | 3.375 | 3.565 | 3.935 |
| ES16 | Ethyl palmitate | 28.268 | MS, RI | 16.234 | 17.389 | 18.365 | 19.397 | 20.23 | 21.765 | 24.567 | 18.218 | 19.624 | 20.253 |
| ES17 | Ethyl linoleate | 31.429 | MS, RI | 12.756 | 13.655 | 14.363 | 16.876 | 13.658 | 15.655 | 18.765 | 12.264 | 14.158 | 17.931 |
|  | Σ |  |  | 67.193 | 71.194 | 75.658 | 83.72 | 87.41 | 93.605 | 104.349 | 74.898 | 79.798 | 87.891 |
|  | *Alcohols* |  |  |  |  |  |  |  |  |  |  |  |  |
| AL1 | 3-ethoxy-1-propanol | 1.358 | MS, RI | ND | 0.264 | 0.378 | 0.558 | 0.615 | 0.744 | 0.798 | 0.607 | 0.663 | 0.715 |
| AL2 | 3-methyl-butanol | 5.583 | MS, RI | 0.491 | 0.581 | 0.567 | 0.508 | 0.627 | 0.607 | 0.556 | 0.552 | 0.533 | 0.516 |
| AL3 | Isoamyl alcohol | 5.842 | MS, RI | 0.129 | 3.365 | 3.034 | 3.035 | 3.755 | 3.623 | 3.552 | 2.975 | 2.670 | 2.123 |
| AL4 | 1-hexanol | 7.281 | MS, RI | ND | 0.883 | 0.838 | 0.825 | 0.832 | 0.904 | 0.975 | 0.809 | 0.843 | 0.921 |
| AL5 | 2-methyl-1-propanol | 10.321 | MS, RI | ND | 1.365 | 1.576 | 1.658 | 1.332 | 1.656 | 1.851 | 0.975 | 1.242 | 1.565 |
| AL6 | 1-octen-3-ol | 11.11 | MS, RI | 0.311 | 0.553 | 0.466 | 0.421 | 0.552 | 0.501 | 0.434 | 0.432 | 0.372 | 0.334 |
| AL7 | Enanthol | 11.223 | MS, RI | ND | 0.276 | 0.295 | 0.301 | 0.224 | 0.287 | 0.338 | 0.203 | 0.242 | 0.310 |
| AL8 | Isooctanol | 12.107 | MS, RI | ND | 0.438 | 0.453 | 0.501 | 0.513 | 0.643 | 0.862 | 0.487 | 0.586 | 0.785 |
| AL9 | 2,3-butanediol | 13.399 | MS, RI | 4.470 | 4.534 | 4.715 | 4.974 | 4.860 | 5.162 | 5.323 | 4.601 | 4.983 | 5.024 |
| AL10 | Octanol | 13.611 | MS, RI | ND | 0.518 | 0.495 | 0.489 | 0.517 | 0.512 | 0.503 | 0.418 | 0.445 | 0.443 |
| AL11 | 1-nonanol | 15.966 | MS, RI | ND | 0.313 | 0.354 | 0.386 | 0.411 | 0.473 | 0.502 | 0.378 | 0.391 | 0.423 |
| AL12 | 2-Furanmethanol | 16.124 | MS, RI | 0.686 | 0.838 | 0.835 | 0.821 | 0.851 | 0.846 | 0.843 | 0.802 | 0.771 | 0.769 |
| AL13 | Benzyl Alcohol | 20.635 | MS, RI | 1.232 | 1.656 | 1.453 | 1.265 | 1.886 | 1.556 | 1.466 | 1.415 | 1.398 | 1.303 |
| AL14 | Phenylethyl alcohol | 21.304 | MS, RI | 14.903 | 16.356 | 18.778 | 20.694 | 20.367 | 23.355 | 27.378 | 17.536 | 19.389 | 20.472 |
|  | Σ |  |  | 22.222 | 31.94 | 34.237 | 36.436 | 37.342 | 40.869 | 45.381 | 32.19 | 34.528 | 35.703 |
|  | *Aldehydes* |  |  |  |  |  |  |  |  |  |  |  |  |
| AD1 | 2-Heptenal | 7.882 | MS, RI | ND | 0.439 | 0.427 | 0.411 | 0.839 | 0.846 | 0.854 | 0.448 | 0.438 | 0.454 |
| AD2 | Nonaldehyde | 9.445 | MS, RI | ND | 0.228 | 0.233 | 0.241 | 0.362 | 0.377 | 0.380 | 0.158 | 0.169 | 0.174 |
| AD3 | Benzaldehyde | 12.705 | MS, RI | 1.947 | 2.221 | 2.143 | 2.365 | 2.492 | 2.596 | 2.525 | 1.527 | 1.576 | 1.486 |
| AD4 | Benzeneacetaldehyde | 15.487 | MS, RI | 0.477 | 0.541 | 0.514 | 0.513 | 0.646 | 0.667 | 0.666 | 0.441 | 0.464 | 0.457 |
| AD5 | 2-undecenal | 17.696 | MS, RI | ND | 0.153 | 0.125 | 0.094 | 0.130 | 0.104 | 0.085 | 0.068 | 0.045 | 0.038 |
| AD6 | Pentanal | 18.153 | MS, RI | ND | 0.484 | 0.462 | 0.451 | 0.679 | 0.679 | 0.688 | 0.534 | 0.573 | 0.288 |
| AD7 | 2-phenyl-2-butenal | 21.581 | MS, RI | 1.576 | 1.784 | 1.734 | 1.687 | 1.994 | 2.114 | 2.197 | 1.698 | 1.874 | 1.976 |
| AD8 | 2-Pyrrolecarbaldehyde | 23.613 | MS, RI | 0.919 | 1.022 | 0.985 | 0.975 | 1.231 | 1.232 | 1.248 | 0.962 | 0.952 | 0.952 |
|  | Σ |  |  | 4.919 | 6.872 | 6.623 | 6.737 | 8.373 | 8.615 | 8.643 | 5.836 | 6.091 | 5.825 |
|  | *Ketones* |  |  |  |  |  |  |  |  |  |  |  |  |
| KE1 | 2-octanone | 6.962 | MS, RI | 3.547 | 5.675 | 5.786 | 5.765 | 8.043 | 8.098 | 8.087 | 6.014 | 5.752 | 5.321 |
| KE2 | 2-nonanone | 9.352 | MS, RI | ND | 0.356 | 0.275 | 0.234 | 0.450 | 0.332 | 0.263 | 0.283 | 0.189 | 0.153 |
| KE3 | 3-hydroxy-2-butanone (Acetoin) | 9.611 | MS, RI | ND | 0.368 | 0.334 | 0.333 | 0.635 | 0.657 | 0.655 | 0.254 | 0.235 | 0.204 |
|  | Σ |  |  | 3.547 | 6.399 | 6.395 | 6.332 | 9.128 | 9.087 | 9.005 | 6.551 | 6.176 | 5.678 |
|  | *Alkanes* |  |  |  |  |  |  |  |  |  |  |  |  |
| AK1 | Tetramethylethylene | 4.321 | MS, RI | ND | 0.287 | 0.238 | 0.136 | 0.354 | 0.345 | 0.340 | 0.121 | 0.096 | 0.145 |
| AK2 | Decamethylcyclopentasiloxane | 4.781 | MS, RI | 0.339 | 0.458 | 0.524 | 0.598 | 0.616 | 0.732 | 0.869 | 0.304 | 0.321 | 0.432 |
| AK3 | Dodecamethylcyclohexasiloxane | 8.819 | MS, RI | 0.602 | 0.768 | 0.731 | 0.722 | 0.811 | 0.817 | 0.832 | 0.628 | 0.517 | 0.437 |
| AK4 | Tetradecane | 9.658 | MS, RI | 0.421 | 0.438 | 0.454 | 0.488 | 0.491 | 0.523 | 0.646 | 0.332 | 0.387 | 0.402 |
| AK5 | Pentadecane | 12.017 | MS, RI | 1.056 | 1.159 | 1.216 | 1.257 | 1.335 | 1.650 | 1.836 | 0.607 | 0.738 | 1.013 |
| AK6 | Caryophyllene | 14.099 | MS, RI | 2.493 | 3.537 | 3.753 | 3.981 | 4.571 | 4.743 | 5.113 | 3.045 | 4.055 | 4.178 |
| AK7 | Hexadecane | 14.333 | MS, RI | 0.802 | 1.078 | 1.027 | 0.928 | 1.087 | 1.074 | 1.121 | 0.668 | 0.556 | 0.468 |
| AK8 | α-Caryophyllene | 15.727 | MS, RI | 0.195 | 0.423 | 0.435 | 0.438 | 0.454 | 0.451 | 0.442 | 0.294 | 0.352 | 0.362 |
| AK9 | Octamethylcyclotetrasiloxane | 21.032 | MS, RI | 0.674 | 0.681 | 0.690 | 0.702 | 0.709 | 0.734 | 0.785 | 0.685 | 0.692 | 0.703 |
|  | Σ |  |  | 6.582 | 8.829 | 9.068 | 9.25 | 10.428 | 11.069 | 11.984 | 6.684 | 7.714 | 8.14 |
|  | *Volatile phenols* |  |  |  |  |  |  |  |  |  |  |  |  |
| PH1 | 4-Vinylphenol | 20.312 | MS, RI | 0.678 | 0.642 | 0.638 | 0.621 | 0.635 | 0.641 | 0.649 | 0.365 | 0.398 | 0.325 |
| PH2 | 4-Vinyl guaiacol | 23.345 | MS, RI | 0.568 | 0.534 | 0.538 | 0.548 | 0.551 | 0.548 | 0.552 | 0.276 | 0.287 | 0.223 |
| PH3 | 2-Methoxy-4-vinylphenol | 26.754 | MS, RI | 1.532 | 1.524 | 1.515 | 1.502 | 1.476 | 1.486 | 1.511 | 0.554 | 0.452 | 0.365 |
|  | Σ |  |  | 2.778 | 2.7 | 2.691 | 2.671 | 2.662 | 2.675 | 2.712 | 1.195 | 1.137 | 0.913 |
|  | *Pyrazine* |  |  |  |  |  |  |  |  |  |  |  |  |
| PY1 | 2-methylpyrazine | 6.769 | MS, RI | 0.238 | 0.314 | 0.351 | 0.391 | 0.425 | 0.467 | 0.520 | 0.375 | 0.405 | 0.423 |
| PY2 | 2,6-dimethylpyrazine | 8.1 | MS, RI | 0.239 | 0.286 | 0.334 | 0.352 | 0.379 | 0.468 | 0.557 | 0.312 | 0.352 | 0.401 |
| PY3 | 2,3,5-trimethylpyrazine | 9.797 | MS, RI | 0.439 | 0.498 | 0.523 | 0.587 | 0.608 | 0.643 | 0.719 | 0.468 | 0.487 | 0.620 |
|  | Σ |  |  | 0.916 | 1.098 | 1.208 | 1.33 | 1.412 | 1.578 | 1.796 | 1.155 | 1.244 | 1.444 |
|  | *Pyrrole* |  |  |  |  |  |  |  |  |  |  |  |  |
| PYR1 | 2-acetylpyrrole | 22.559 | MS, RI | 1.195 | 1.281 | 1.314 | 1.354 | 1.352 | 1.553 | 1.822 | 1.217 | 1.302 | 1.353 |
|  | Σ |  |  | 1.195 | 1.281 | 1.314 | 1.354 | 1.352 | 1.553 | 1.822 | 1.217 | 1.302 | 1.353 |
|  | *Furan* |  |  |  |  |  |  |  |  |  |  |  |  |
| FU1 | 2-Hexanoylfuran | 16.512 | MS, RI | 1.853 | 1.898 | 1.922 | 1.962 | 2.151 | 2.556 | 2.987 | 1.807 | 2.056 | 2.294 |
|  | Σ |  |  | 1.853 | 1.898 | 1.922 | 1.962 | 2.151 | 2.556 | 2.987 | 1.807 | 2.056 | 2.294 |
